# Immunization with Complete Freund’s Adjuvant Reveals Trained Immunity-like Features in A/J Mice

**DOI:** 10.3390/vaccines13070768

**Published:** 2025-07-21

**Authors:** Kiruthiga Mone, Shraddha Singh, Fatema Abdullatif, Meghna Sur, Mahima T. Rasquinha, Javier Seravalli, Denise K. Zinniel, Indranil Mukhopadhyay, Raul G. Barletta, Teklab Gebregiworgis, Jay Reddy

**Affiliations:** 1 School of Veterinary Medicine and Biomedical Sciences, University of Nebraska-Lincoln, Lincoln, NE 68583, USA; kmone2@huskers.unl.edu (K.M.); ssingh22@huskers.unl.edu (S.S.); mesur@coh.org (M.S.); mahima.rasquinha@mssm.edu (M.T.R.); dzinniel2@unl.edu (D.K.Z.); rbarletta@unl.edu (R.G.B.); 2 Department of Biochemistry, Schulich School of Medicine and Dentistry, University of Western Ontario, London, ON N6A 5C1, Canada; fabdull7@uwo.ca; 3 Department of Immuno-Oncology, Beckmann Research Institute, City of Hope, Duarte, CA 91010, USA; 4 Department of Immunology and Immunotherapy, Icahn School of Medicine at Mount Sinai, New York, NY 10029, USA; 5 Department of Biochemistry, University of Nebraska-Lincoln, Lincoln, NE 68588, USA; 6 Department of Statistics, University of Nebraska-Lincoln, Lincoln, NE 68583, USA; imukhopadhyay2@unl.edu; 7 Department of Oncology, Schulich School of Medicine and Dentistry, University of Western Ontario, London, ON N6A 5W9, Canada

**Keywords:** trained immunity, adjuvants, Freund’s adjuvants, complete Freund’s adjuvant, incomplete Freund’s adjuvant, CFA, IFA, metabolomics, epigenetics, innate immune memory, vaccine adjuvants, immunometabolism

## Abstract

Background/Objectives: Freund’s adjuvants induce different immunomodulatory effects, but their underlying molecular mechanisms are unclear. In this study, we investigated whether the immune-stimulating effects of the complete Freund’s adjuvant (CFA) involve the mechanisms of trained immunity (TI). Methods: We examined bone marrow cells (BMCs) isolated from CFA-immunized A/J mice to address this question. Incomplete Freund’s adjuvant (IFA) and *Mycobacterium tuberculosis* var. *bovis* Bacillus Calmette-Guérin (BCG) served as negative and positive controls, respectively. We evaluated cytokine profiles, metabolic, and epigenetic changes. Results: First, BMCs from all groups except saline showed varied levels of IL-1β, IL-6, and TNF-α. But expression of CCL5 and CXCL10 was significantly elevated only in the CFA and BCG groups. Transcriptionally, significant elevations were noted for TNF-α and IL-1β in the CFA and BCG groups, whereas CXCL10, IL-6, and IL-10 were upregulated in the CFA and BCG groups, respectively. Second, while BMCs from the BCG group expressed the markers of both the M1 and M2 macrophages, no clear trends were noted in the CFA and IFA groups. Third, cell lysates from the CFA group revealed metabolic reprogramming in the BMCs. Specifically, we observed an increased level of lactate, indicative of aerobic glycolysis, which is implicated in TI, and this was also detected in the IFA group. Fourth, epigenetic analysis revealed histone enrichment in the promoter region of TNF-α, in the CFA group, but to a lesser degree than the BCG group. However, no epigenetic changes were observed in the IFA group. Conclusions: Our data provide new insights into the mechanisms of Freund’s adjuvants and the immunomodulatory effects of CFA could involve the features of TI.

## 1. Introduction

Adjuvants are traditionally used to enhance immune responses to vaccines. Although adjuvants are not antigens, their ability to activate innate leukocytes is critical for the induction of adaptive immune responses [1]. Historically, Freund’s adjuvants have been used in experimental research, and their immunomodulatory effects have been best described based on the T helper (Th)1 and Th2 paradigm [2]. The complete Freund’s adjuvant (CFA), a potent adjuvant, promotes mainly a Th1 response, which is ascribed to the *Mycobacterium tuberculosis* (*M. tb*) component [2]. In contrast, the effects of incomplete Freund’s adjuvant (IFA) lacking the *M. tb* extract are shown to be associated with the induction of Th2 cytokines [3]. However, reports indicate that CFA and IFA could induce mixed cytokine profiles with varied amounts [4,5]. Thus, low amounts of Th1 or Th2 cytokines induced by each could influence the immune responses. Thus, determining the cellular and molecular mechanisms of adjuvants might help identify pathways that modulate immune responses.

Both CFA and IFA contain a similar oily base of mannide monooleate [6]. Because of the mycobacterial content, CFA induces a robust interferon (IFN)-γ-producing Th1 response [2], but it can also promote Th17 responses [7,8]. These aspects are also well studied with various live mycobacterial strains, including *M. tb* var. *bovis* Bacillus Calmette-Guérin (BCG) [9], a known inducer of trained immunity (TI) [10,11,12,13,14].

The concept of TI has gained significant attention in vaccine research [15,16,17]. Essentially, the innate leukocytes, predominantly macrophages primed with BCG or β-glucan as primary stimuli, show a heightened response to the primary as well as the secondary stimuli [10,11,18]. Because BCG and CFA contain similar mycobacterial content, we sought to investigate whether the effects mediated by CFA could involve the mechanisms of TI. Additionally, in an infection study with Coxsackievirus B3 in A/J mice, we noted that animals immunized with CFA were protected from infection [19], suggesting that the CFA effects might involve the induction of TI. To test our hypothesis, we immunized mice with CFA and BCG using IFA and saline as control groups. After four weeks, bone marrow cells (BMCs) were isolated and stimulated with or without lipopolysaccharide (LPS) as a secondary stimulus. The data revealed that CFA immunization led to alterations in the metabolic features suggestive of TI, but the histone enrichment was noted moderately in the promoter region of tumor necrosis factor (TNF)-α. Since IFA and CFA are used extensively in experimental research and they display varied effects, our data provide further insights as to their underlying mechanisms.

## 2. Materials and Methods

### 2.1. Mice

A/J mice (6 to 8 weeks old) were obtained from the Jackson Laboratory (Bar Harbor, ME, USA). The animals were housed according to the guidelines of the Institutional Animal Care and Use Committee at the University of Nebraska-Lincoln (protocol #2321). BCG immunization studies were conducted under biosafety level 2 conditions. Mice were euthanized using a carbon dioxide chamber following the recommendations of the Panel on Euthanasia of the American Veterinary Medical Association.

### 2.2. BCG Propagation and Enumeration

In this study, *Mycobacterium tuberculosis* var. *bovis* BCG str. Pasteur 1173P2 was used, as previously stated, with some modifications [20]. This strain was inoculated from a starter culture at an initial optical density (OD)_600_ of 0.05. The bacteria were grown in Middlebrook 7H9 broth/10% Oleic Acid-Albumin-Dextrose-Catalase for 4 days at 100 rpm to the late exponential phase (OD_600_ of 1.25). The culture was centrifuged and resuspended in freezing media for storage at −80 °C. Frozen BCG aliquots were thawed, washed twice, and resuspended in phosphate-buffered saline (PBS). To prevent clumping, the suspension was passed through a 26-gauge needle ten times prior to administration in mice.

### 2.3. Study Design

Immunizations were carried out using IFA and CFA (Sigma-Aldrich, St. Louis, MO, USA). CFA supplemented with heat-killed *M. tb* H37Ra extract (Difco Laboratories, Detroit, MI, USA) to a final concentration of 5mg/mL was used for immunizations [21,22,23,24,25]. As indicated above, we made an observation that the A/J mice immunized with the *M. tb*-enriched CFA were protected from infection [19]. Hence, we sought to use the same composition for consistency. Groups of animals (*n* = 3/group) were immunized subcutaneously on day 0 with 200 µL of saline, IFA, or CFA emulsions into the shoulder and hip regions. BCG was administered intravenously at a dose of (5 × 10^6^) colony-forming units/animal, with one animal receiving a partial amount subcutaneously. At the study endpoint on day 28, the animals were euthanized, and blood was collected by cardiac puncture. BMCs were harvested for further experiments (Supplementary Figure S1).

### 2.4. Bone Marrow Cells Isolation and Ex Vivo Stimulation

BMCs were isolated from the forelimbs and hindlimbs by flushing the bones with Roswell Park Memorial Institute (RPMI) 1640 medium using a 27-gauge needle. The cell suspension was filtered through a 70-µm cell strainer to remove debris and used for downstream analysis. BMCs were stimulated with or without 10 ng/mL LPS for 24 h as a secondary stimulus. In these experiments, we used LPS as a secondary stimulus that has been routinely used to capture the events of TI [10,18,26,27,28]. Following stimulation, supernatants and cell lysates were collected for cytokine bead array and real-time quantitative PCR (RT-qPCR) analysis, respectively. Cell lysates from unstimulated samples were processed for metabolomic and epigenetic analyses and stored at –80 °C until further use (Supplementary Figure S1).

### 2.5. Cytokine Bead Array Analysis

Supernatants collected 24 h post-LPS stimulation of BMCs were used for cytokine bead array analysis. Cytokines and chemokines were measured using the LEGENDplex™ Mouse Anti-Virus Response Panel (13-plex; BioLegend, San Diego, CA, USA). These include IFN-γ, CXCL1 (KC), TNF-α, CCL2 (MCP-1), IL-12p70, CCL5 (RANTES), IL-1β, CXCL10 (IP-10), GM-CSF, IL-10, IFN-β, IFN-α, and IL-6. The lyophilized cytokine standard mix provided in the kit was serially diluted to generate a standard curve. Test samples and diluted standards were incubated with capture beads/cytokine antibody conjugates, followed by the addition of detection antibodies and streptavidin–phycoerythrin reagents. Beads were acquired using flow cytometry, and cytokine concentrations were quantified using the LEGENDplex™ Data Analysis Software Suite, Version 2025-05-01 (BioLegend) [29].

### 2.6. RT-qPCR

Bone marrow cell lysates collected 24 h post-LPS stimulation were used for RT-qPCR analysis. Total RNA was isolated using the PureLink™ RNA Mini Kit (Invitrogen, Waltham, MA, USA), treated with deoxyribonuclease I, and quantified using a NanoDrop™ ND-1000 spectrophotometer (Thermo Fisher Scientific, Waltham, MA, USA). RNA was reverse-transcribed and amplified in a single-step reaction using the iTaq Universal SYBR Green One-Step kit (Bio-Rad, Hercules, CA, USA). Using the CFX96 Real-Time PCR Detection System (Bio-Rad), qPCR analysis was performed for TNF-α, IL-6, IL-10, IL-1β, CCL2, CCL5, and CXCL10, with GAPDH as a control. Similarly, expression of F4/80, peroxisome proliferator-activated receptor gamma (PPAR-γ), CD206, nitric oxide synthase 2 (NOS2), CD80, and CD86 was also analyzed. Primer sequences used are listed in Supplementary Table S1. Cytokine expression levels were normalized to GAPDH using the 2^−^(^∆∆^Ct) method [29].

### 2.7. Targeted Metabolomics

Cell lysates (5 × 10^6^ cells each) were thawed on ice and suspended in 500 μL of lysis solution (80% methanol and 20% water, spiked with 2.13 μM ^13^C_5_-^15^N_1_-proline and 12 μM ^13^C_6_-glucose as internal standards). Approximately 50 mg of 0.5 Zirconium oxide beads (0.5 mm, Next Advance, Troy, NY, USA) were then added. The cells were disrupted by 3 cycles at a power setting of 8 and at 4 °C. The samples were then centrifuged at 14,000× *g* for 10 min at 4 °C. The supernatants were transferred into a new 2.0 mL Eppendorf tubes and evaporated down to a pellet using a Speedvac centrifuge (Fisher Scientific, Hampton, NH, USA). The pellets with metabolites were stored at −70 °C until the liquid chromatography coupled with high-resolution mass spectrometry (LC-MS) analysis was performed. A Sciex 4000 QTrap (Sciex, Framingham, MA, USA) attached to an Agilent (Santa Clara, CA, USA) LC-1200 high-performance liquid chromatography (HPLC) system was used to measure the polar metabolites concentrations. The mobile phase A was LC-Grade acetonitrile while mobile phase B was 20 mM ammonium hydroxide, 20 mM ammonium acetate pH 9.50 prepared in LC-Grade water. The column used for the separation used a hydrophilic interaction liquid chromatography (HILIC) type of stationary phase, an Amide X-Bridge (4.6 × 100 mm, Waters, Milford, MA, USA) column operating at a flow rate of 0.5 mL/min at 20 °C. The gradient consisted of 5% mobile phase B for 3 min, followed by a linear gradient from 5 to 70% mobile phase B over 20 min, 70% to 95% mobile phase B over 1 min, 95% B for 4 min, switch from 95% to 5% B over 1 min and finally re-equilibration with 5% mobile phase B for 9 min. The triple quadrupole instrument was operated in MRM (multiple reaction monitoring) mode in positive and negative ionization modes, with the Q1/Q3 transitions [30]. The stored metabolite pellets were suspended in 50 μL of LC-Grade water at 4 °C. After vortexing briefly, samples were pipetted into 300 μL polyethylene vials with crimp seal tops. All samples were maintained at 5 °C in the autosampler throughout the analysis. Conditions for the electrospray ionization source were as follows: temperature, 550 °C; curtain gas, 20 psi; ion source potential, +5000 V or −4200 V; declustering potentials set at +50 or −60 volts; ionization Gas1 or Gas2, 70 psi. An injection volume of 10 μL was used throughout. The data files were imported into Metaboanalyst 3.0 (Sciex, Framingham, MA, USA), which was used to extract the individual compound (MRMs) chromatograms for all the samples and standard mixtures. The compound areas were exported into MS-Excel and converted into pmoles per million cells based on external calibration curves for each compound, sample volume, and the number of cells. The data were then exported as *csv files for further chemometric analysis.

### 2.8. Metabolomics Data Analysis

To study the structure of the dataset and assess the difference between the groups, both pairwise heatmaps and volcano plots were generated using R (version 4.4.1) [31]. Heatmaps were plotted using the pheatmap package [32]. Data normalizations were performed by rows to normalize metabolite levels across samples, and hierarchical clustering was applied using Ward’s method to group similar profiles [33]. Pairwise comparisons of individual metabolites were conducted using unpaired Student’s *t*-test in GraphPad Prism (version 10.4.2). Outlier detection was performed using Dixon’s Q test, using R (version 4.4.1) [31,34]. Relative changes in metabolites between groups are presented by normalizing the values of the groups under investigation to the mean of the reference group. Pathway analysis was performed using MetaboAnalyst version 6 [35] for metabolites that showed significant differences between groups as determined by Student’s *t*-test.

### 2.9. Chromatin Immunoprecipitation (ChIP)-qPCR Assay

Bone marrow cells (1 × 10^7^) were fixed with 37% formaldehyde to a final concentration of 1% for 10 min at room temperature with rotation to crosslink protein–DNA interactions. Crosslinking was quenched by adding 2.5 mL of 1.25 M ice-cold glycine, followed by incubation on ice for 5 min. Cells were centrifuged at 300× *g* for 5 min at 4 °C and washed three times with ice-cold PBS. Cell pellets were resuspended in 1 mL of nuclei isolation buffer containing protease inhibitors, incubated on ice for 10 min, and centrifuged at 3000 rpm for 5 min at 4 °C. Supernatants were discarded, and the resulting nuclei pellets were snap-frozen in liquid nitrogen and stored at –80 °C until further use. For chromatin extraction, frozen pellets were thawed and lysed in 330 µL of sodium dodecyl sulfate lysis buffer, followed by incubation on ice for 20 min. Chromatin was sheared using a Bioruptor Pico sonicator (Diagenode, Denville, NJ, USA) for 10 cycles (30 s ON, 30 s OFF). Samples were centrifuged at 12,000× *g* for 10 min at 4 °C, and the resulting supernatant containing sheared chromatin was collected. A 45 μL aliquot of chromatin was used per immunoprecipitation (IP), and 4.5 μL of the sheared samples was used as input control (10%).

For IP, 30 µL of Protein A Dynabeads (Thermo Fisher Scientific) were washed twice with PBS containing Tween-20. The beads were incubated overnight at 4 °C with 2.5 µg of histone 3 trimethylation of lysine 4 (H3K4me3) antibody or isotype control (IgG) (Diagenode). After antibody binding, beads were washed twice with PBS containing Tween-20, resuspended in ChIP dilution buffer, and incubated with 45 µL of sheared chromatin overnight at 4 °C with rotation. The DNA–protein complexes were sequentially washed for 5 min each with low-salt, high-salt, and lithium chloride wash buffers, followed by two washes with tris-ethylenediaminetetraacetic acid (TE) buffer. Chromatin was eluted in 100 μL of elution buffer at 65 °C with rotation overnight. Eluates and input samples were treated with RNase A in TE buffer at 37 °C for 30 min. Reverse crosslinking was performed at 55 °C for 1 h by adding 5 M NaCl, 0.5M EDTA (pH 8.0), 1M Tris (pH 7.2), and proteinase K. DNA was purified using the QIAquick MinElute PCR Purification Kit (Qiagen, Hilden, Germany). Purified DNA was quantified using a Qubit 4 fluorometer (Thermo Fisher Scientific), and ChIP-qPCR was performed using SYBR green-based amplification (Universal SYBR Green Master Mix, Bio-Rad) for TNF-α, IL-6, IL-1β, and IL-10. GAPDH and myoglobin (Diagenode) served as positive and negative controls, respectively. Primer sequences used are listed in Supplementary Table S2. Results were expressed as the percentage of input DNA.

### 2.10. Statistical Analysis

Statistical analyses were conducted using R (version 4.4.1) [31]. Normality of all datasets was assessed using the Kolmogorov–Smirnov test. For cytokine bead array and RT-qPCR datasets, multiple comparisons were performed using one-way Analysis of Variance (ANOVA), followed by pairwise comparisons using a *t*-test. For epigenetic datasets, two-way ANOVA, and one-way ANOVA were used for multiple comparisons, and a *t*-test was used for pairwise, and within-group comparisons.

## 3. Results and Discussion

### 3.1. CFA and BCG Immunizations Were Associated with Distinct Cytokine and Chemokine Profiles Compared to IFA

**Figure 1:**
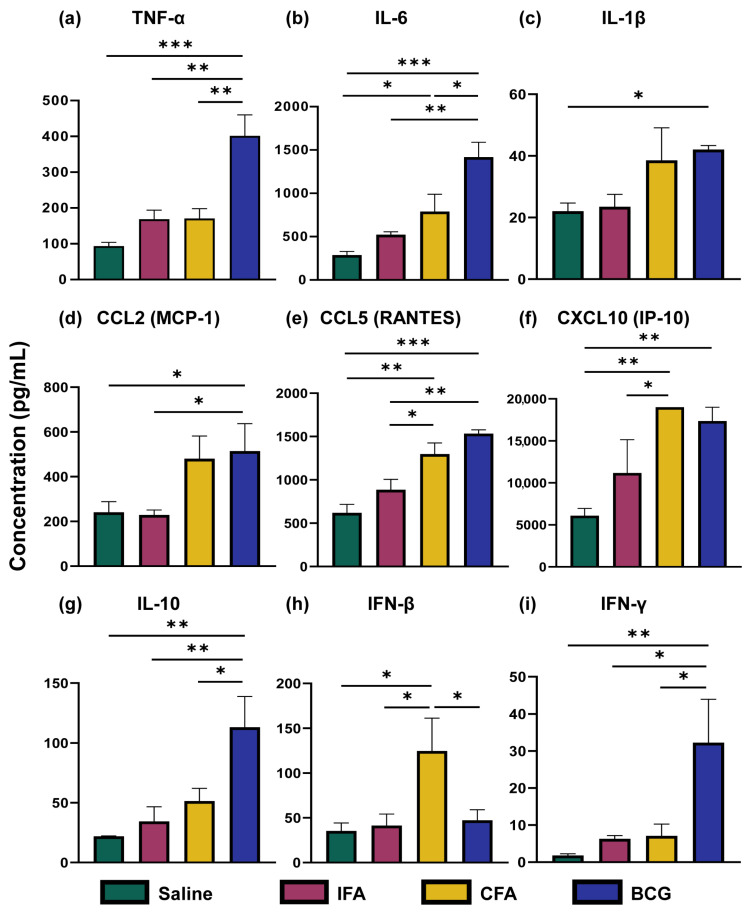
Cytokine and chemokine analysis in mice immunized with various adjuvants. BMCs harvested on day 28 from mice injected with saline, IFA, CFA, or BCG were stimulated ex vivo with LPS (10 ng/mL) for 24 h. Cytokine and chemokine levels in the culture supernatants were quantified using a LEGENDplex™ cytokine bead array (BioLegend), as described in the Methods section. Panel labels (**a**–**i**) indicate the specific cytokines or chemokines measured: (**a**) TNF-α, (**b**) IL-6, (**c**) IL-1β, (**d**) CCL2 (MCP-1), (**e**) CCL5 (RANTES), (**f**) CXCL10 (IP-10), (**g**) IL-10, (**h**) IFN-β, and (**i**) IFN-γ. The x-axis indicates the different treatment groups, saline (green), IFA (red), CFA (yellow), and BCG (blue), and the y-axis indicates cytokine and chemokine concentrations (pg/mL). Data are represented as mean ± SEM (*n* = 3 per group). Statistical significance was determined using an unpaired *t*-test. * *p* < 0.05, ** *p* < 0.01, *** *p* < 0.001. TNF, Tumor necrosis factor; IL, Interleukin; CCL, C-C motif chemokine ligand; CXCL, C-X-C motif chemokine ligand; IFN, Interferon.

First, we analyzed the culture supernatants of BMCs for innate cytokines and chemokines, including IFN-γ and IL-10 (anti-inflammatory cytokine), using multiplex bead array analysis (Figure 1a–i). The data revealed elevated levels of IL-6 (*p* = 0.030), CCL5 (*p* = 0.0016), CXCL-10 (*p* = 0.0031), and IFN-β (*p* = 0.016) in the CFA group as compared to saline-treated controls. In contrast, the BCG group showed elevations with a broad range of cytokines [TNF-α (*p* = 0.00027), IL-6 (*p* = 0.00036), IL-1β (*p* = 0.042), CCL2 (*p* = 0.0497), CCL5 (*p* = 0.00024), CXCL-10 (*p* = 0.0065), IL-10 (*p* = 0.0028), and IFN-γ (*p* = 0.0077)], but not IFN-β as compared to the saline group. Importantly, none of the tested cytokines and chemokines were significantly increased in the IFA group, suggesting its weak immunostimulatory effects. Furthermore, the elevated levels of CCL5 (*p* = 0.022), CXCL-10 (*p* = 0.035), and IFN-β (*p* = 0.022) in CFA relative to the IFA group could be due to the presence of killed *M. tb* extract. The elevated TNF-α (*p* = 0.0016), IL-6 (*p* = 0.0016), CCL2 (*p* = 0.043), CCL5 (*p* = 0.0021), IL-10 (*p* = 0.0064), and IFN-γ (*p* = 0.017) levels in BCG as compared to IFA group highlights the sustained immune effects of the live BCG. However, no significant alterations were noted with CXCL1, IL-12p70, GM-CSF, and IFN-α.

We then evaluated cytokine expression at the transcription level by RT-qPCR using the BMCs derived from various treatment groups. The data revealed that the TNF-α (*p* = 0.024), IL-1β (*p* = 0.046), and CXCL10 (*p* = 0.040) were upregulated in the CFA group compared to the saline group (Supplementary Figure S2). Similarly, significant upregulation of TNF-α (*p* = 0.011), IL-6 (*p* = 0.028), IL-1β (*p* = 0.037), and IL-10 (*p* = 0.018) expression was noted in the BCG group as compared to the saline group. Conversely, only IL-1β (*p* = 0.011) and IL-10 (*p* = 0.045) were elevated in the IFA group compared to saline recipients. However, no significant differences were noted with any of the cytokines tested when compared between CFA and BCG or CFA and IFA groups (Supplementary Figure S2). The discrepancies noted between protein and mRNA levels suggest that transcription and secretion do not always correlate. Overall, similar cytokine patterns as noted with TNF-α, IL-6, IL-1β, CCL5, and CXCL-10 between CFA and BCG groups suggest common immune activation pathways [14,26,36,37,38,39].

### 3.2. Immunizations with Freund’s Adjuvants Did Not Reveal Differences in the Expression of M1 and M2 Markers in BMCs

**Figure 2:**
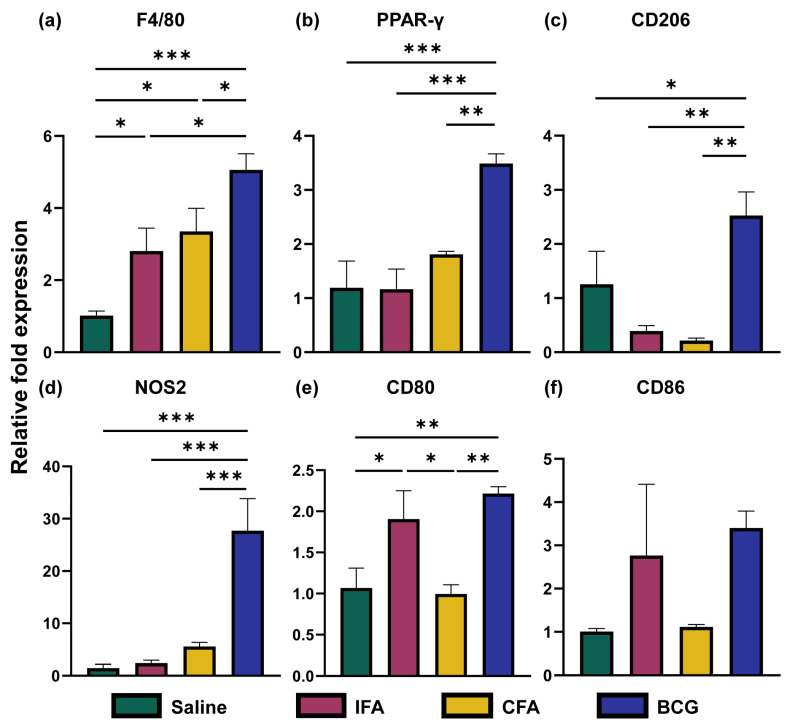
Phenotypic analysis of macrophages. Groups of mice were administered with saline, IFA, CFA, or BCG, and BMCs were isolated on day 28. BMCs were stimulated ex vivo with LPS (10 ng/mL) for 24 h, after which total RNA was extracted and analyzed by RT-qPCR for M1 and M2 macrophage markers. Panel labels (**a**–**f**) indicate the specific markers assessed: (**a**) F4/80, (**b**) PPAR-γ, (**c**) CD206, (**d**) NOS2, (**e**) CD80, and (**f**) CD86. Gene expression levels were normalized to GAPDH, and relative expressions were calculated using the 2^−^(^∆∆^Ct) method. The x-axis indicates the different treatment groups, saline (green), IFA (red), CFA (yellow), and BCG (blue), and the y-axis indicates the relative fold expression. Data are presented as mean ± SEM (*n* = 3 per group). Statistical significance was determined using an unpaired *t*-test. * *p* < 0.05, ** *p* < 0.01, *** *p* < 0.001. PPAR-γ, Peroxisome Proliferator-Activated Receptor-γ; NOS2, Nitric Oxide Synthase 2.

Generally, macrophages are classified into M1 and M2 subsets, representing pro- and anti-inflammatory functionalities, respectively [40,41]. M1 and M2 macrophages represent classically and alternatively activated macrophages, respectively [42]. To investigate these aspects, we measured the expression of F4/80, the marker of macrophages, and the expression of M1 (NOS2, CD80, and CD86) and M2 (PPAR-γ and CD206) markers by RT-qPCR (Figure 2a–f). We noted that the F4/80 expression was increased in the BMCs of all treatment groups (IFA (*p* = 0.038), CFA (*p* = 0.012), and BCG (*p* = 0.0005)) compared to the saline group. But the highest expression level of F4/80 was noted in the BCG group. Next, by analyzing the M1 markers, we noted the expression of NOS2 only in the BCG group (*p* = 0.00035), although a trend was noted in the CFA group. However, the expression of costimulatory molecules showed variations. While CD80 expression was increased in the BCG (*p* = 0.0065) and IFA groups (*p* = 0.029), its downregulated expression in the CFA compared to the IFA group was not expected. A similar trend was also noted for CD86. As for M2 markers, expression of both PPAR-γ and CD206 was upregulated only in the BCG group (*p* = 0.0011, *p* = 0.045) compared to other groups. Taken together, the data suggest that the macrophage phenotypes did not alter in the adjuvant groups, whereas those of BCG were mixed as previously reported [18,43].

### 3.3. CFA Immunization Was Associated with Metabolic Reprogramming in the BMCs

Immune cell metabolism plays a critical role in regulating immune activation and function [44]. Metabolites serve as energy sources and building blocks for macromolecules, and act as signaling molecules and epigenetic modulators [45,46]. Immune cell activation and altered function are associated with metabolic reprogramming [47,48]. Metabolite alterations in response to adjuvants are gaining attention, which could contribute to their adjuvanticity [49].

**Figure 3:**
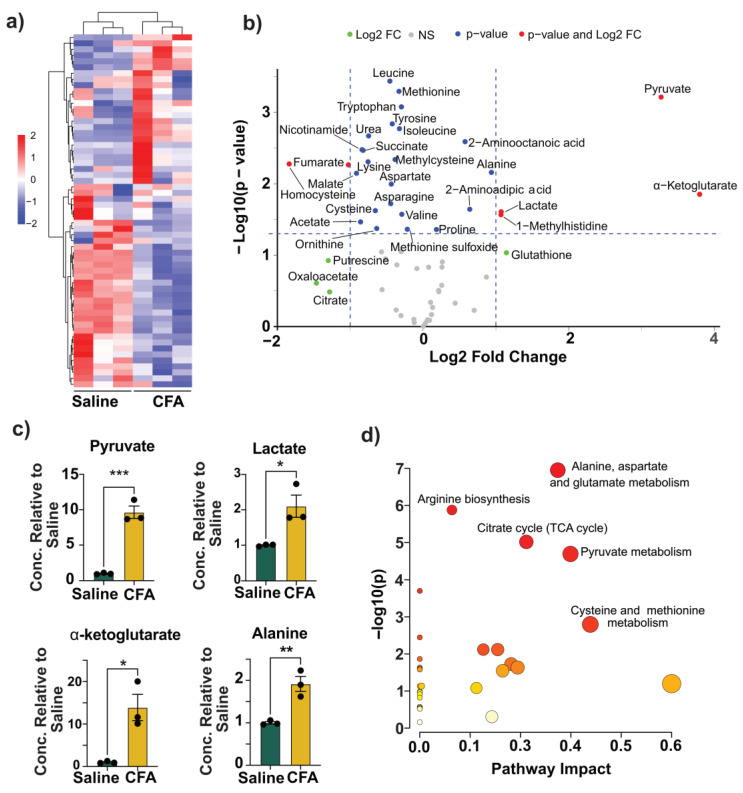
Metabolic alterations in BMCs from mice immunized with CFA. (**a**) Heatmap with hierarchical clustering of metabolites between saline- and CFA-treated groups. Each row represents a metabolite, and each column corresponds to a sample. The color scale indicates relative metabolite abundance (red: higher, blue: lower), normalized across samples. Ward’s clustering method was applied to both metabolites and samples, revealing distinct clustering patterns. (**b**) Volcano plot showing differential metabolite abundance. Metabolites with |log_2_FC| > 1 and –log_10_(*p*-value) > 1.3 are highlighted. (**c**) Relative concentrations of the indicated metabolites in the saline and CFA groups. (**d**) Pathway enrichment analysis was generated for significantly altered metabolites obtained by comparing the metabolite levels in saline and CFA groups. The analysis was conducted using metabolites that showed statistically significant differences (*p* < 0.05) between the CFA and saline groups. Pathways were mapped using MetaboAnalyst 6.0, and enrichment scores reflect both the significance and the pathway impact based on topology analysis. The highlighted pathways include pyruvate metabolism, the TCA cycle, alanine, aspartate and glutamate metabolism, arginine biosynthesis, and cysteine and methionine metabolism. Each pathway node represents the relative contribution of individual metabolites and is scaled by both statistical significance and pathway centrality. Data are shown as mean ± SEM; *n* = 3 per group. * *p* < 0.05, ** *p* < 0.01, *** *p* < 0.001 by an unpaired two-tailed *t*-test.

Recently, by evaluating systemic metabolic responses to Freund’s adjuvants, we reported that the serum metabolite profiles of CFA and BCG were closely aligned [20]. This similarity suggests that the CFA could exert immunomodulatory effects, which may involve alterations in the metabolites of TI similar to BCG [20,28,50,51]. We performed a targeted mass spectrometry analysis on BMC lysates to assess metabolic changes, by quantifying 59 metabolites across different pathways (Supplementary Table S3) [52,53]. These included the metabolites of the end products of glycolysis, intermediates of the tricarboxylic acid (TCA) cycle, amino acids and their derivatives, antioxidants involved in the maintenance of redox balance, and methylation/choline pathway metabolites. We compared the metabolite profiles of the CFA-treated and the control (saline-treated) group using hierarchical clustering analysis by means of the Ward clustering method. The resulting heatmap revealed a clear separation between the two groups, indicating globally distinct metabolic signatures (Figure 3a and Supplementary Figure S3). Metabolites were clustered into two primary branches, one comprising 25 metabolites elevated in the CFA group, and the other, 35 metabolites with unchanged or reduced levels. Within the elevated branch, a distinct subcluster included lactate, pyruvate, phenylalanine, homocysteine, 2-aminoadipic acid, and 2-aminooctanoic acid. Conversely, a separate subcluster of reduced metabolites in the CFA group included oxalate, itaconate, glycine, isocitrate, and citrate (Supplementary Figure S3). We further visualized differential metabolite abundance using a volcano plot, plotting log_2_ fold change (FC) against −log_10_ *p*-values (Figure 3b). Pyruvate, lactate, α-ketoglutarate, and 1-methylhistidine were significantly elevated in the CFA group (log_2_FC > 1, *p* < 0.05), while homocysteine and fumarate were significantly decreased. Additional 21 metabolites met the *p*-value cutoff but not the FC threshold, while seven others exceeded the FC threshold without statistical significance. Our data suggest that CFA injection alters key metabolic pathways, particularly glycolysis, the TCA cycle, and amino acid metabolism. To further interrogate these changes, we focused on the end products of glycolysis, pyruvate, and lactate, as indicators of glycolytic activity. It is well established that the TI is associated with the Warburg effect indicated by increased aerobic glycolysis involving pyruvate conversion to lactate in the presence of oxygen [28,50,51,54]. We noted elevated levels of both pyruvate and lactate in the CFA group (Figure 3c), suggesting enhanced glycolytic flux consistent with the metabolic rewiring associated with TI [28,50,51].

Next, we examined the levels of the TCA cycle intermediates, including malate, fumarate, succinate, and α-ketoglutarate. CFA-treated BMCs showed decreased levels of malate, fumarate, and succinate, along with a significant increase in α-ketoglutarate (Figure 3c, Supplementary Figure S4a). While these findings do not conform to the reported metabolic features of TI, it is possible that concurrent accumulation of α-ketoglutarate may reflect increased glutaminolysis [27,28,51]. In addition to lactate conversion, pyruvate can also be transaminated into alanine via alanine transaminase, generating α-ketoglutarate as a byproduct [55]. In our study, CFA-treated BMCs showed increased levels of alanine, indicating that pyruvate could also be diverted into this transamination pathway (Figure 3c). This likely contributes to the observed rise in α-ketoglutarate, supporting that the CFA immunization induces a metabolic shift favoring both lactate and alanine production (Figure 3c). We also observed a significant reduction in aspartate levels in the CFA group relative to the saline control (Supplementary Figure S4a). Overall, the decrease in aspartate, and the TCA intermediates could suggest a downregulation of the TCA cycle activity or redirection of these metabolites into other biosynthetic pathways.

We further performed pathway enrichment analysis using metabolites that were significantly altered between the CFA and saline groups (*p* < 0.05; Supplementary Table S4). The data revealed enrichment of several core pathways, including alanine, aspartate, and glutamate metabolism, the TCA cycle, and pyruvate metabolism. Additionally, pathways related to arginine biosynthesis and cysteine and methionine metabolism were also significantly impacted (Figure 3d). These observations may point to a possibility of broad rewiring of amino acid and energy metabolisms. In cellular metabolism, arginine can be degraded by arginase-1 to produce ornithine and urea [56]. In our study, both ornithine and urea levels were significantly reduced in the CFA group, although arginine levels remained unchanged (Supplementary Figure S4b). Additionally, aspartate, which combines with citrulline to form arginosuccinate, a precursor of arginine, and fumarate, a byproduct of this pathway, were also decreased, as described earlier (Supplementary Figure S4a). This pattern is consistent with reduced arginase activity, as M1-like macrophages could have a diversion in the arginine metabolism away from the urea cycle and toward nitric oxide production [57,58,59]. Our findings suggest that CFA may promote a shift in arginine metabolism that could support immune effector functions [60]. Methionine serves as a precursor for S-adenosylmethionine, a universal methyl donor involved in epigenetic regulation [61,62]. However, our data revealed a reduction in methionine levels in the CFA group which may be due to its increased consumption for methylation processes (Supplementary Figure S4c). The decreased homocysteine and cysteine levels may indicate their enhanced use for maintaining redox balance via glutathione synthesis (Supplementary Figure S4c) [63,64]. Our data revealed such a trend with glutathione levels (Supplementary Figure S4d). Together, the data support the idea that CFA could induce metabolic alterations resembling a few features of TI, including a shift toward aerobic glycolysis (the Warburg effect).

### 3.4. Immunization with BCG Reveals Metabolic Changes That Overlap with CFA

**Figure 4:**
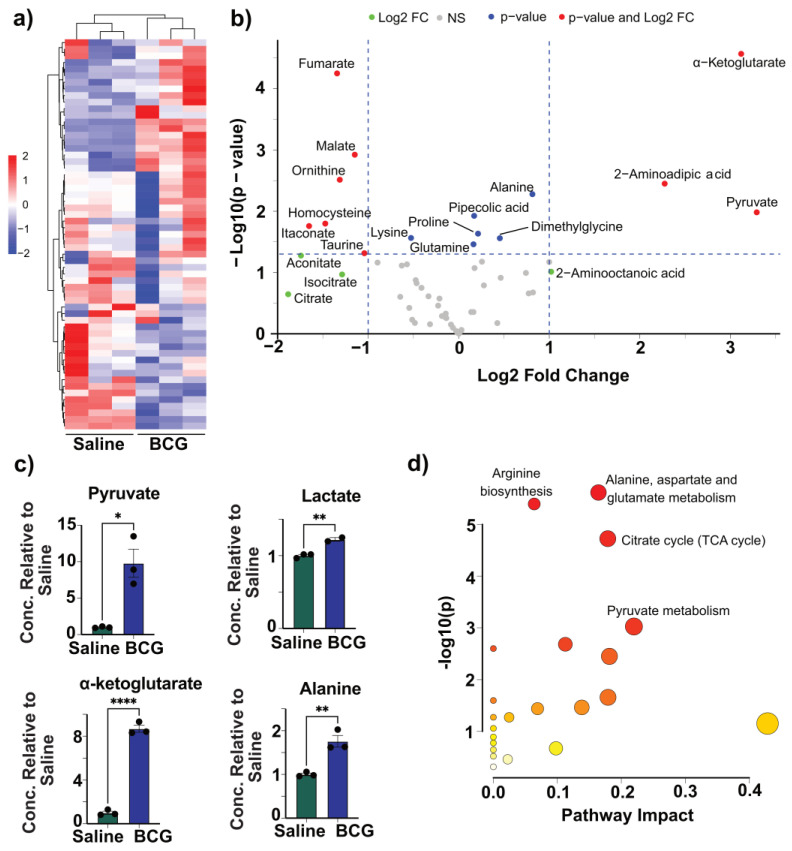
Metabolic alterations in BMCs from mice immunized with BCG. (**a**) Heatmap with hierarchical clustering of metabolites between saline- and BCG-treated groups. Each row represents a metabolite, and each column corresponds to a sample. The color scale indicates relative metabolite abundance (red: higher, blue: lower), normalized across samples. Ward’s clustering method was applied to both metabolites and samples, revealing distinct clustering patterns. (**b**) Volcano plot showing differential metabolite abundance. Metabolites with |log_2_FC| > 1 and −log_10_(*p*-value) > 1.3 are highlighted. (**c**) Relative concentrations of the indicated metabolites in the saline and BCG groups. (**d**) Pathway enrichment analysis was generated for significantly altered metabolites obtained by comparing the metabolite levels in saline and BCG groups. The analysis was conducted using metabolites that showed statistically significant differences (*p* < 0.05) between the BCG and saline groups. Pathways were mapped using MetaboAnalyst 6.0, and enrichment scores reflect both the significance and the pathway impact based on topology analysis. The key enriched pathways include pyruvate metabolism, the TCA cycle, alanine, aspartate and glutamate metabolism, and arginine biosynthesis. Each node in the pathway diagram reflects the relative importance and statistical contribution of metabolites within each pathway. Data are shown as mean ± SEM; *n* = 3 per group. * *p* < 0.05, ** *p* < 0.01, **** *p* < 0.0001 by an unpaired two-tailed *t*-test.

Given that CFA-induced metabolic features partially resemble TI, we next compared its metabolic profile to that of BCG, a well-established inducer of this phenomenon [13,28]. Using the same analytical pipeline, we first evaluated the overall metabolite patterns in BCG- and saline-treated groups. Hierarchical clustering (Ward’s method) revealed two distinct clusters corresponding to BCG and saline groups, indicating a clear separation of their metabolic signatures (Figure 4a and Supplementary Figure S5). Clustering of metabolites formed two major branches, which further divided into multiple smaller clusters, suggesting a broad and complex perturbation in response to BCG. We then generated a volcano plot to identify significantly altered metabolites in the BCG group. Pyruvate, α-ketoglutarate, and 2-aminoadipic acid were significantly elevated (log_2_FC > 1, *p* < 0.05), while six metabolites, fumarate, malate, ornithine, homocysteine, itaconate, and taurine, were significantly decreased (Figure 4b). An additional set of six metabolites, including alanine, pipecolic acid, proline, lysine, glutamine, and dimethylglycine, showed significant changes (*p* < 0.05) but with smaller fold changes (log_2_FC < 1). Notably, the increased levels of pyruvate and α-ketoglutarate in the BCG group mirrored the pattern observed in CFA-treated samples (Figure 4b), suggesting shared metabolic hallmarks. Further analysis of glycolysis-related metabolites showed elevated levels of pyruvate in the BCG group, with lactate also trending upward (Figure 4c and Supplementary Figure S6). One sample displayed markedly higher lactate levels than the others; applying Dixon’s test confirmed it as an outlier. After correction too, lactate levels were consistently elevated in the BCG group (Supplementary Figure S6). Similar to CFA, BCG-treated BMCs showed reduced levels of the TCA intermediates, malate, and fumarate, alongside increased α-ketoglutarate. However, unlike the CFA group, succinate levels remained unchanged in the BCG group, which was not expected (Figure 4c and Supplementary Figure S7a). We also observed increased levels of alanine, whereas aspartate, a direct product of oxaloacetate, was unaffected by BCG, as opposed to its reduction in the CFA group (Figure 4c and Supplementary Figure S7a). We then performed enrichment analysis using the metabolites significantly altered between the BCG and saline groups (Supplementary Table S5). Similar to CFA, BCG immunization influenced several pathways. These include alanine, aspartate, and glutamate metabolism, the TCA cycle, pyruvate metabolism, and arginine biosynthesis (Figure 4d). While the affected pathways overlapped between BCG and CFA, the direction and magnitude of metabolite changes varied. For example, the BCG group showed a slight increase in glutamine and a decrease in ornithine, with urea and arginine levels remaining unchanged (Supplementary Figure S7b). Although methionine and cysteine levels were unchanged in the BCG group, similar to CFA, homocysteine was reduced (Supplementary Figure S7c). While alterations, such as in the TCA intermediates do not conform to the canonical features of BCG-induced TI [27,28,51], the bacterial strains used in different studies could contribute to variations [26,65,66]. Taken together, our data suggest that CFA and BCG could involve similar metabolic pathways linked to TI, particularly aerobic glycolysis. However, their metabolic fingerprints diverge, with CFA prominently affecting sulfur amino acid metabolism.

### 3.5. IFA Induces Modest Changes to Glycolysis

**Figure 5:**
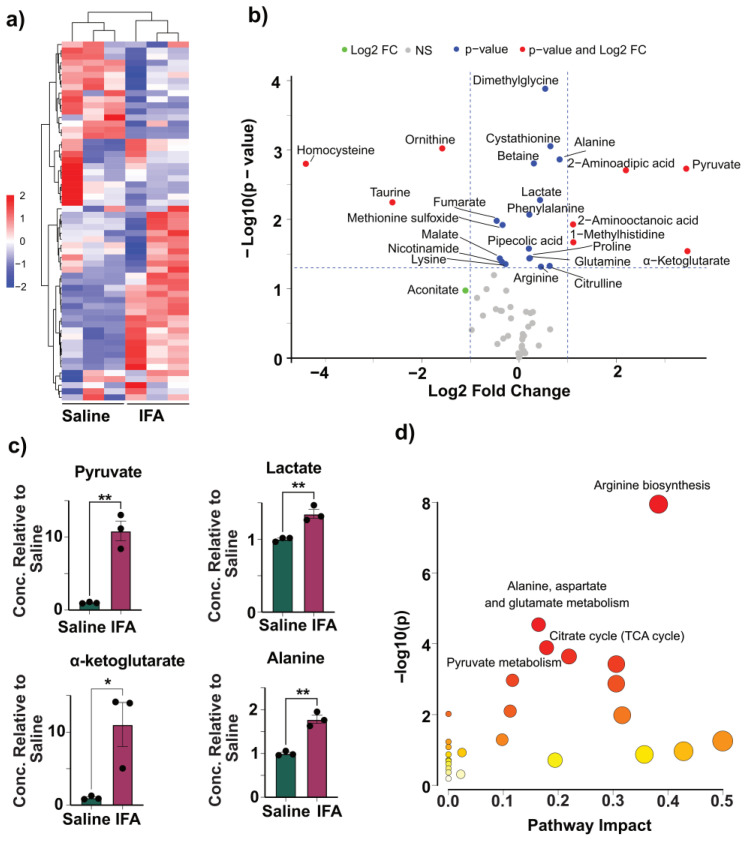
IFA immunization induces metabolic reprogramming in BMCs similar to CFA but with arginine biosynthesis as the major impacted pathway. (**a**) Heatmap with hierarchical clustering of metabolites between saline- and IFA-treated groups. Each row represents a metabolite, and each column corresponds to a sample. The color scale indicates relative metabolite abundance (red: higher, blue: lower), normalized across samples. Ward’s clustering method was applied to both metabolites and samples, revealing distinct clustering patterns. (**b**) Volcano plot showing differential metabolite abundance. Metabolites with |log_2_FC| > 1 and −log_10_(*p*-value) > 1.3 are highlighted. (**c**) Relative concentrations of the indicated metabolites in the saline and IFA groups. (**d**) Pathway enrichment analysis was generated for significantly altered metabolites obtained by comparing the metabolite levels in saline and IFA groups. The analysis was conducted using metabolites that showed statistically significant differences (*p* < 0.05) between the IFA and saline groups. Pathways were mapped using MetaboAnalyst 6.0, and enrichment scores reflect both the significance and the pathway impact based on topology analysis. The prominently enriched pathway was arginine biosynthesis, followed by pyruvate metabolism, the TCA cycle, and alanine, aspartate, and glutamate metabolism. Each node in the pathway diagram reflects the relative importance and statistical contribution of metabolites within each pathway. Data are shown as mean ± SEM; *n* = 3 per group. * *p* < 0.05, ** *p* < 0.01, by an unpaired two-tailed *t*-test.

To assess the metabolic effects of IFA, which contains only the surfactant mannide monooleate in mineral oil, we performed targeted mass spectrometry similar to the CFA and BCG groups. Heatmap analysis revealed that the IFA and saline groups clustered separately, indicating a distinct metabolic profile for IFA-treated cells (Figure 5a and Supplementary Figure S8). Volcano plot analysis identified five significantly increased metabolites in the IFA group: pyruvate, α-ketoglutarate, 2-aminoadipic acid, 2-aminooctanoic acid, and 1-methylhistidine. In contrast, levels of ornithine, taurine, and homocysteine were significantly decreased (Figure 5b). An additional 15 metabolites, including dimethylglycine, cystathionine, alanine, lactate, betaine, phenylalanine, fumarate, malate, pipecolic acid, citrulline, proline, and lysine, were also altered in the IFA group, but their log_2_FC values were less than 1. Despite these modest changes, a slight increase in lactate and alanine levels were observed, suggesting a limited glycolytic response similar in direction, though weaker, to that seen in CFA and BCG groups (Figure 5c). The lactate-to-pyruvate ratio was 86.5 in the IFA group compared to 149.4 in the CFA group (Supplementary Figure S9). This indicates a more pronounced conversion of pyruvate to lactate in CFA-treated cells, suggesting stronger glycolytic flux. Reductions in the TCA cycle intermediates malate and fumarate, along with an increase in α-ketoglutarate, were also noted in the IFA group, mirroring trends seen in CFA and BCG groups (Figure 5c and Supplementary Figure S10a). However, unlike CFA-treated cells, the levels of succinate and aspartate remained unchanged in IFA-treated cells (Supplementary Figure S10a). Pathway enrichment analysis revealed that arginine biosynthesis was the most prominently affected pathway in the IFA group. Other pathways include alanine, aspartate, and glutamate metabolism, the TCA cycle, and pyruvate metabolism (Figure 5d and Supplementary Table S6). Within the arginine biosynthesis pathway, ornithine and urea were decreased, whereas citrulline and arginine levels were elevated compared to the saline group (Supplementary Figure S10b).

To identify differences between CFA and IFA, we visualized the metabolites using a volcano plot, leading us to note changes mainly in the amino acids (Supplementary Figure S11). Next, by conducting a pairwise comparison, we observed a broad downregulation of amino acids in the CFA group as compared to the IFA group. These include phenylalanine, tyrosine, tryptophan, cysteine, cystathionine, valine, isoleucine, and lysine (Supplementary Figure S12a). In contrast, homocysteine and taurine amino acids and itaconate were significantly increased in the CFA group (Supplementary Figure S12b). Additionally, the TCA cycle intermediates (oxaloacetate and succinate) were decreased in the CFA group (Supplementary Figure S12c). Overall, alterations noted in the amino acids may have a role in balancing the pro- and anti-inflammatory properties of adjuvants [67,68]. For example, taurine that has been previously implicated in TI can modulate inflammatory functions similar to homocysteine [69,70].

### 3.6. CFA Immunization Reveals Minor Epigenetic Changes

**Figure 6:**
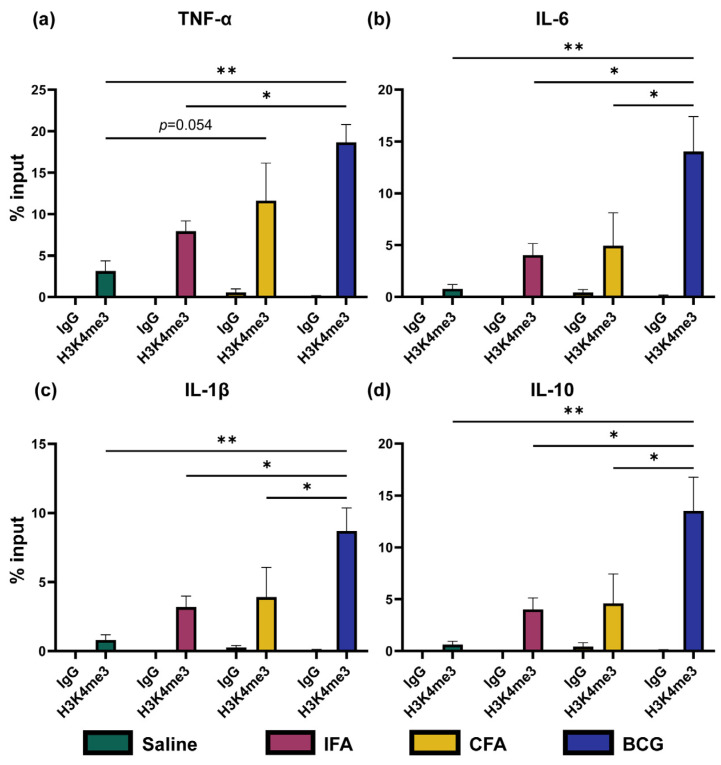
Histone enrichment analysis in BMCs derived from mice immunized with various adjuvants. BMCs isolated from mice administered with saline, IFA, CFA, or BCG on day 28 were processed for ChIP-qPCR. Chromatin was crosslinked with 1% formaldehyde, and ChIP was performed using an antibody specific for H3K4me3 or the isotype control IgG, followed by DNA purification and qPCR using primers targeting promoter regions of (**a**) TNF-α, (**b**) IL-6, (**c**) IL-1β, and (**d**) IL-10. The x-axis indicates the different treatment groups, saline (green), IFA (red), CFA (yellow), and BCG (blue) treated with H3K4me3 or isotype IgG control antibodies, and the y-axis indicates the histone enrichment represented as % of input DNA. Data are presented as mean ± SEM (*n* = 3 per group). Statistical significance was determined using an unpaired *t*-test. * *p* < 0.05, ** *p* < 0.01. TNF, Tumor necrosis factor; IL, Interleukin.

To determine the epigenetic changes, we performed ChIP-qPCR analysis using IgG as an antibody control and myoglobin as a negative control [10,71]. Essentially, enrichment in the histone methylation in the promoter regions of pro-inflammatory cytokines, TNF-α, IL-6, and IL-1β is considered a hallmark of TI induced by BCG [10]. Specifically, H3K4me3 represents an open chromatin and active gene transcription [72,73]. In our analysis, we tested for histone deposition, H3K4me3, in the inflammatory cytokines indicated above, in addition to IL-10. Expectedly, BMCs from the BCG group revealed a significant increase in the H3K4me3 mark in TNF- α (*p* = 0.0034), IL-6 (*p* = 0.0044), IL-1β (*p* = 0.0046), and IL-10 (*p* = 0.0036) as compared to other groups. However, histone enrichment in IL-10 was not expected (Figure 6a–d). It may be that the IL-10 could balance the transcriptional activity in shifting the chromatin toward a regulatory state, still requiring histone remodeling and turnover. Unexpectedly, we also noted histone enrichment in the myoglobin region in the BCG group. We did not capture similar profiles in the CFA group when compared with the saline recipients, except for TNF-α (*p* = 0.054). We determined epigenetic changes at a relatively longer time point (day 28 post-immunizations). Whether their determinations at earlier time points could facilitate the capture of other signature cytokines, such as IL-6 and IL-1β, requires additional studies [74]. But such short-term studies may not yield information on the sustained responses. However, in a similar analysis for the IFA group, the data did not reveal differences for any cytokines when compared with the saline group. It is possible that CFA and BCG both contain *M. tb* components that strongly activate innate immune pathways, including Toll-like receptor signaling, which has been shown to induce TI [75,76]. The enrichment of H3K4me3 at the TNF-α promoter but not in other cytokines in the CFA group may also be due to the nature of the mycobacterial component (CFA, killed vs. BCG, live). It is to be noted that BCG is a live attenuated strain of *M. tb* var.* bovis*, that can persist in host cells, inducing a robust and sustained immune response [76,77,78]. In contrast, CFA contains heat-killed *M. tb* extract emulsified in non-metabolizable paraffin oil with the surfactant mannide monooleate. These compositional differences could have immunological consequences. Additionally, the mineral oil in CFA can cause local inflammation, potentially contributing to nonspecific immune activation. These differences could have contributed to the variations noted between BCG and CFA in our study. In support of this proposition, IFA lacking *M. tb* is a weak inducer of innate activation, and the absence of H3K4me3 enrichments for cytokines underscores the requirement of mycobacterial components for inducing epigenetic changes.

Overall, our integrated analysis of cytokine profiles, macrophage phenotypes, metabolic, and epigenetic changes revealed five major observations. (a) In response to LPS simulation, CFA-treated BMCs exhibited increased secretion of IL-6, CCL5, CXCL10, and IFN-β. However, a broad panel of cytokines (TNF-α, IL-6, IL-1β, CCL2, CCL5, CXCL10, IL-10, and IFN-γ) were noted in the BCG group. The production of inflammatory cytokines TNF-α, IL-6, and IL-1β is a hallmark of TI, and the upregulation of chemokines like CCL2 and CXCL10 further supports this profile, in line with previous reports [26,36,37,38,79]. (b) Interestingly, while BCG induces M1 and M2 cell types, as previously reported [18,43], CFA immunization was not associated with polarized macrophages, suggesting a possibility of an intermediate activation state. (c) On the metabolic level, CFA immunization led to elevated levels of lactate, pyruvate, and alanine, resembling partially the features of TI, but the reduced levels of some TCA intermediates were unexpected [27,28,51]. Although the BCG immunization showed similar metabolite trends, our findings differ slightly from prior studies. These include TCA intermediates, namely, malate, fumarate, and succinate, that were found downregulated [27,28,51]. This discrepancy might be attributed to differences in the BCG strain (e.g., Pasteur in our study vs. Denmark or TICE in others), and mouse strains used (A/J vs. C57Bl/6 or BALB/c) [26,65,66]. Furthermore, most TI studies are performed in humans and/or at earlier time points (day 28 vs. day 7) [28,71,74,80,81]. (d) In contrast, the IFA group revealed no significant change in cytokine production or macrophage polarization. However, its metabolite profiles mirrored some aspects of BCG and CFA-induced metabolic reprogramming. While the traditional view suggests that the effects of CFA stem from its *M. tb* components, our findings suggest that metabolic changes induced by the oil base alone could contribute to immune modulation. The partial overlap in metabolic profiles between CFA and IFA supports this possibility. In addition, we have noted significant alterations with α-ketoglutarate, alanine, fumarate, malate, ornithine, and pyruvate in response to all adjuvants that can modulate innate and adaptive immune responses. For example, α-ketoglutarate is an antioxidant, and it can mediate anti-inflammatory functions and regulate CD4 T cell differentiation [82,83,84,85]. Similarly, alanine is required for normal activation of T cells [86], whereas fumarate possesses antioxidant and anti-inflammatory properties and regulates the production of IL-10 and type I interferons [87,88]. Although less is known about ornithine, malate and pyruvate mediate anti-inflammatory functions, and enhance the effector functions of natural killer cells [89,90]. (e) However, for inducing epigenetic changes, microbial components are critical, as histone enrichments were noted only in the BCG and CFA groups. It is to be noted that the classical markers of BCG-induced TI include increased inflammatory cytokine production (TNF-α, IL-6, and IL-1β), a shift towards aerobic glycolysis with increased lactate, and histone enrichment (H3K4me3) at the promoter regions of TNF-α, IL-6, and IL-1β [13,91,92]. While we captured all of these changes in our studies with BCG, we noted downregulation of malate, fumarate, and succinate, which differed from the previous studies [27,28,51]. Likewise, we partially captured some of the features of TI with CFA immunization. These include cytokine secretion indicated above, histone enrichment at TNF-α, and lactate production (Supplementary Table S7). Similar findings were recently reported with the use of heat-killed *M. tb* [93]. It is possible that these changes could contribute to non-specific protection against infections. For example, we had previously noted that the CFA immunization led to protection against Coxsackievirus B3 infection [19]. Similarly, the active component of CFA (muramyl dipeptide) was shown to confer protection against *Streptococcus pneumoniae* and *Toxoplasma gondii* infections [92,94]. One limitation of this study is the small sample size, which may have reduced the ability to detect more pronounced effects of CFA. A larger sample size could potentially provide clearer insights. Likewise, LPS is routinely used for 24 h as a secondary stimulus to capture the features of TI that we also adapted in our studies [10,18,26,27,28]. Whether evaluations performed at different time points would yield additional information is unclear, which is another limitation. Overall, our data provides new insights into the mechanisms of Freund’s adjuvants, involving metabolic reprogramming, including alterations in amino acid metabolism.

## Data Availability

The original contributions presented in this study are included in the article/Supplementary Material. Further inquiries can be directed to the corresponding authors.

## Supplementary Materials

The following supporting information can be downloaded at: https://www.mdpi.com/article/10.3390/vaccines13070768/s1, Figure S1: The schematic representation of the experimental design; Figure S2: CFA modulates distinct cytokine and chemokine profiles at the transcriptional level; Figure S3: Heatmap showing hierarchical clustering of significantly altered metabolites between saline- and CFA-treated groups; Figure S4: Differential levels of metabolites in saline vs. CFA in BMCs; Figure S5: Heatmap showing hierarchical clustering of significantly altered metabolites between saline- and BCG-treated groups; Figure S6: Lactate concentration in BMCs from the BCG group; Figure S7: Differential levels of metabolites in saline vs. BCG in BMCs; Figure S8: Heatmap showing hierarchical clustering of significantly altered metabolites between saline- and IFA-treated groups; Figure S9: Lactate-to-pyruvate ratio in BMCs from mice immunized with IFA or CFA; Figure S10: Differential level of metabolites in saline vs. IFA in BMCs. Figure S11: Volcano plot illustrating differential metabolite levels between IFA and CFA; Figure S12: Differential level of metabolites in IFA vs. CFA in BMCs; Table S1: Primers used for RT-qPCR [95,96,97,98,99,100]; Table S2: Primers used for ChIP-qPCR [43,101,102]; Table S3: List of metabolites detected and quantified using targeted LC-MS analysis [103]; Table S4: List of metabolites significantly altered in the CFA group compared to the saline group, used to identify major pathways affected by CFA immunization; Table S5: List of metabolites significantly altered in the BCG group compared to the saline group, used to identify major pathways affected by BCG immunization; Table S6: List of metabolites significantly altered in the IFA group compared to the saline group, used to identify major pathways affected by IFA immunization; Table S7: Major findings of the TI features associated with CFA immunization.

## Abbreviations

The following abbreviations are used in this manuscript:

ANOVA	Analysis of Variance
BCG	Bacillus Calmette-Guérin
BMC	Bone marrow cells
CCL	C-C motif chemokine ligand
CFA	Complete Freund’s adjuvant
CXCL	C-X-C motif chemokine ligand
ChIP	Chromatin Immunoprecipitation
FC	Fold change
GAPDH	Glyceraldehyde-3-phosphate dehydrogenase
GM-CSF	Granulocyte-macrophage colony-stimulating factor
HILIC	Hydrophilic interaction liquid chromatography
H3K4me3	Histone 3 trimethylation of lysine 4
HPLC	High-performance liquid chromatography
IFA	Incomplete Freund’s adjuvant
IFN	Interferon
Ig	Immunoglobulin
IL	Interleukin
IP	Immunoprecipitation
LC-MS	Liquid chromatography coupled with high-resolution mass spectrometry
LPS	Lipopolysaccharide
*M. tb*	*Mycobacterium tuberculosis*
MRM	Multiple reaction monitoring
NOS	Nitric oxide synthase
OD	Optical density
PBS	Phosphate-buffered saline
PPAR-γ	Peroxisome proliferator-activated receptor-gamma
RPM	Revolutions per minute
RPMI	Roswell Park Memorial Institute
RT-qPCR	Real-time quantitative polymerase chain reaction
TCA	Tricarboxylic acid
Th	T helper
TI	Trained immunity
TNF	Tumor necrosis factor
TE	Tris-ethylenediaminetetraacetic acid
