# Supplementary material for: Immunization with Complete Freund’s Adjuvant Reveals Trained Immunity-like Features in A/J Mice
Source: Vaccines (Basel). 2025 Jul 21;13(7):768. doi: 10.3390/vaccines13070768 (PMC12300380; doi:10.3390/vaccines13070768)
Supplement: Supplementary file 1 [file vaccines-13-00768-s001.zip › Mone et al_supplementary figures.pdf]

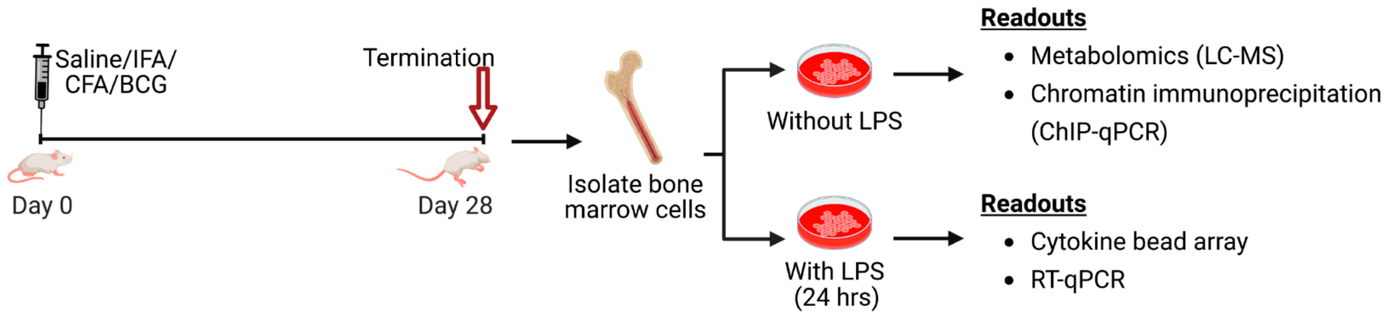

Supplementary Figure S1. The schematic representation of the experimental design. A/J mice were administered with saline, IFA, CFA, or BCG on day 0. On day 28, animals were euthanized, and bone marrow cells were isolated. Cells were stimulated with or without LPS (10 ng/mL) for 24 hours. While, unstimulated cells were processed for metabolomic profiling and chromatin immunoprecipitation/qPCR, LPS-stimulated cells and their supernatants were used for cytokine analysis. Figure created with BioRender.com (accessed on 09 July 2025)

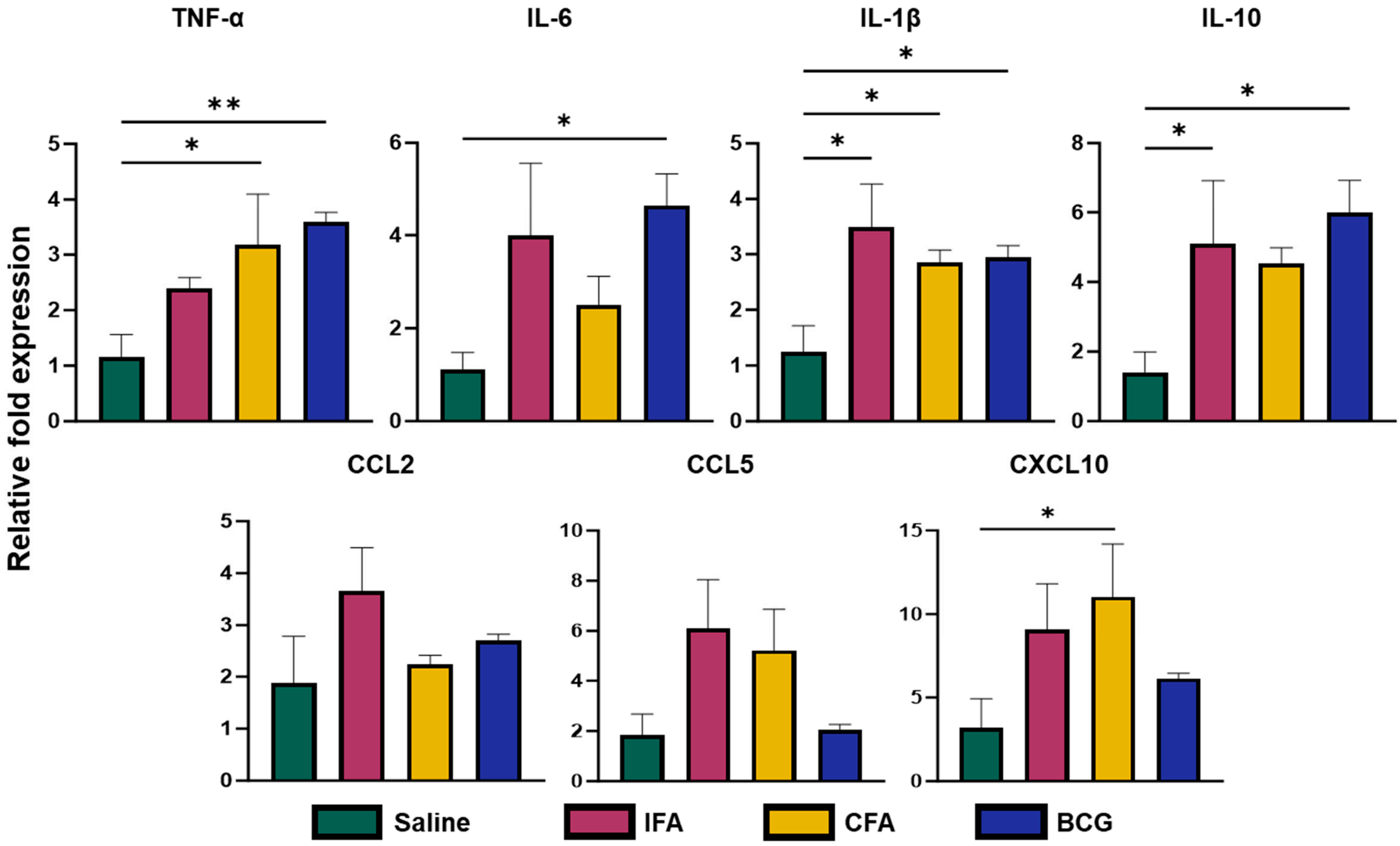

Supplementary Figure S2. CFA modulates distinct cytokine and chemokine profiles at the transcriptional level. BMCs obtained from mice injected with saline, IFA, CFA, or BCG were stimulated ex vivo with LPS for 24 hours. Total RNA was extracted, and RT-qPCR was performed to quantify the expression of indicated cytokines and chemokines. Gene expression levels were normalized to GAPDH, and relative fold changes were calculated using the $2^{-(\Delta\Delta Ct)}$ method. The x-axis indicates the different treatment groups, saline (green), IFA (red), CFA (yellow), and BCG (blue), and the y-axis indicates the relative fold expression. Data are presented as mean ± SEM (n=3 per group). Data are presented as mean ± SEM. Statistical significance was assessed by unpaired t-test. *$p < 0.05$, **$p < 0.01$. TNF, Tumor necrosis factor; IL, Interleukin; CCL, C-C motif chemokine ligand; CXCL, C-X-C motif chemokine ligand; IFN, Interferon.

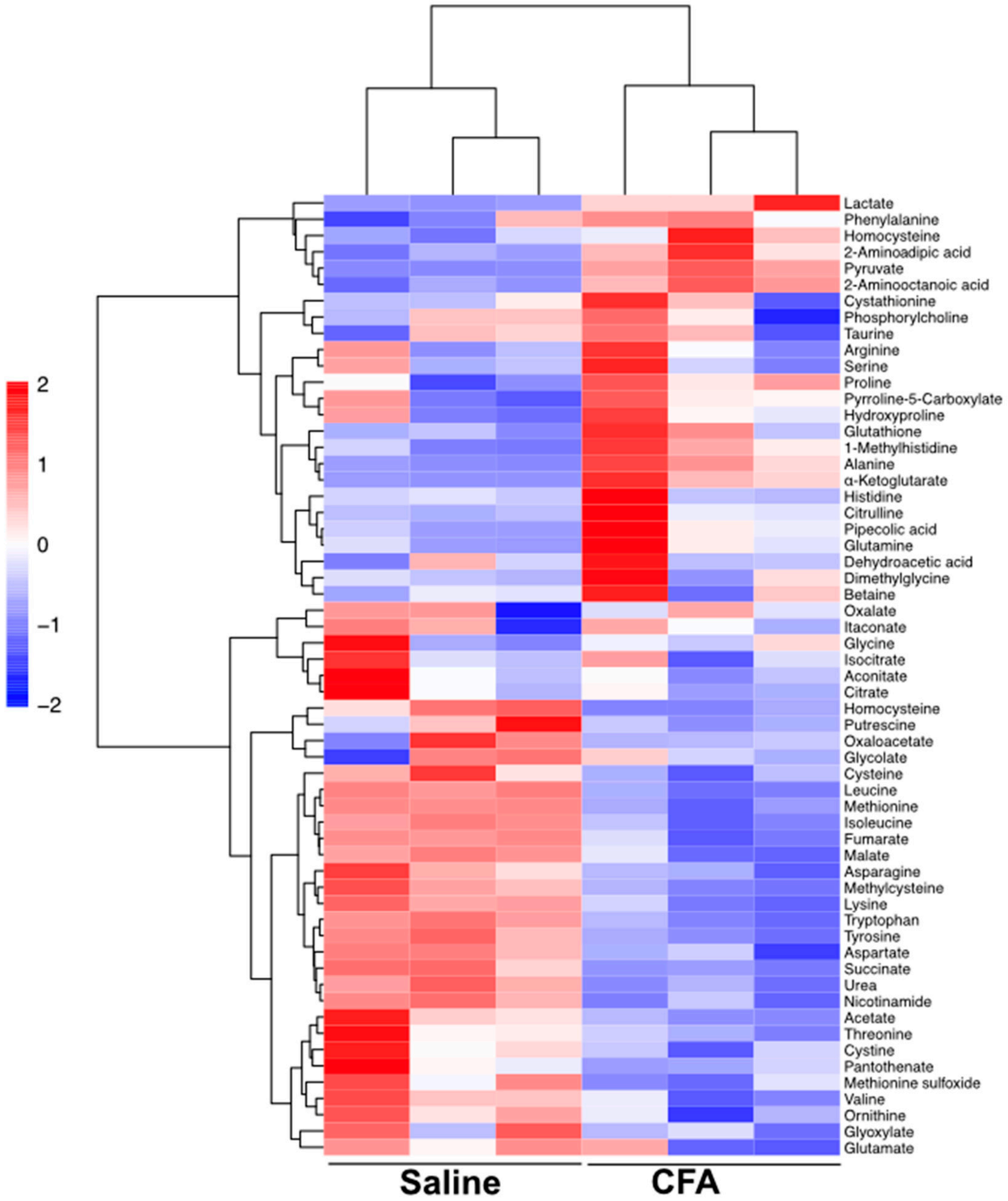

Supplementary Figure S3. Heatmap showing hierarchical clustering of significantly altered metabolites between saline- and CFA-treated groups. Each row represents a metabolite, and each column corresponds to a sample. The color scale indicates relative metabolite abundance (red: higher, blue: lower), normalized across samples. Ward's clustering method was applied to both metabolites and samples, revealing distinct clustering patterns. This analysis highlights marked metabolic differences between the two treatment groups, suggesting that CFA administration induces substantial alterations in metabolic pathways. Group labels are indicated at the bottom of the heatmap, and the clustering dendrogram shows relationships between samples based on metabolic profiles. The dendrogram on the metabolite axis reveals clusters of metabolites that exhibit similar concentration patterns across the samples.

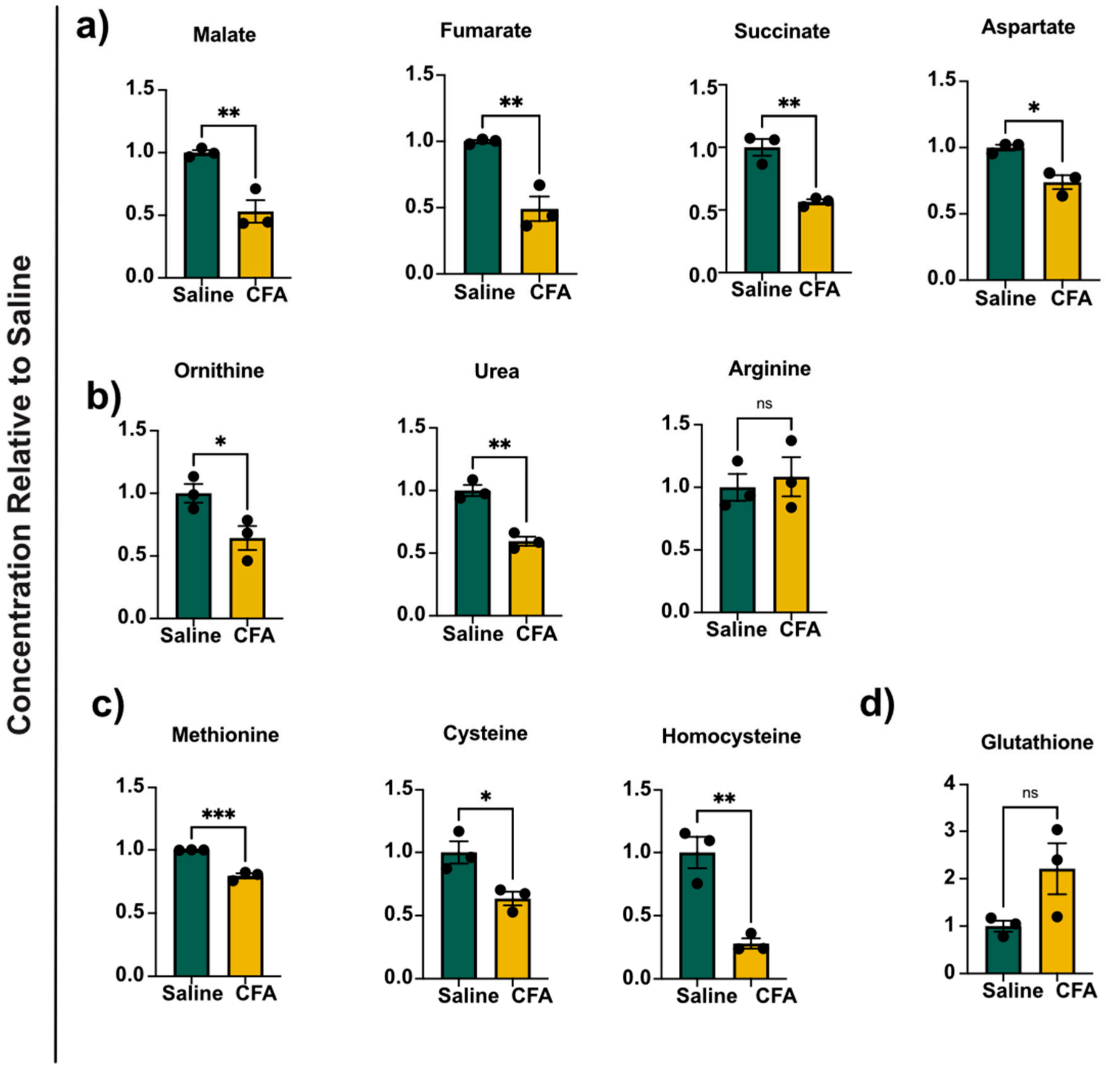

Supplementary Figure S4: Differential levels of metabolites in saline vs. CFA in BMCs. (a) Concentration of metabolites in the TCA cycle, (b) arginine biosynthesis pathway, (c) cysteine and methionine metabolism pathway, and (d) glutathione synthesis pathway. The y-axis indicates relative concentration of metabolites compared with saline, as measured with LC-MS analysis. Data are shown as mean ± SEM; $n = 3$ per group. *$p < 0.05$, **$p < 0.01$, ***$p < 0.001$ by unpaired two-tailed Student's *t*-test.

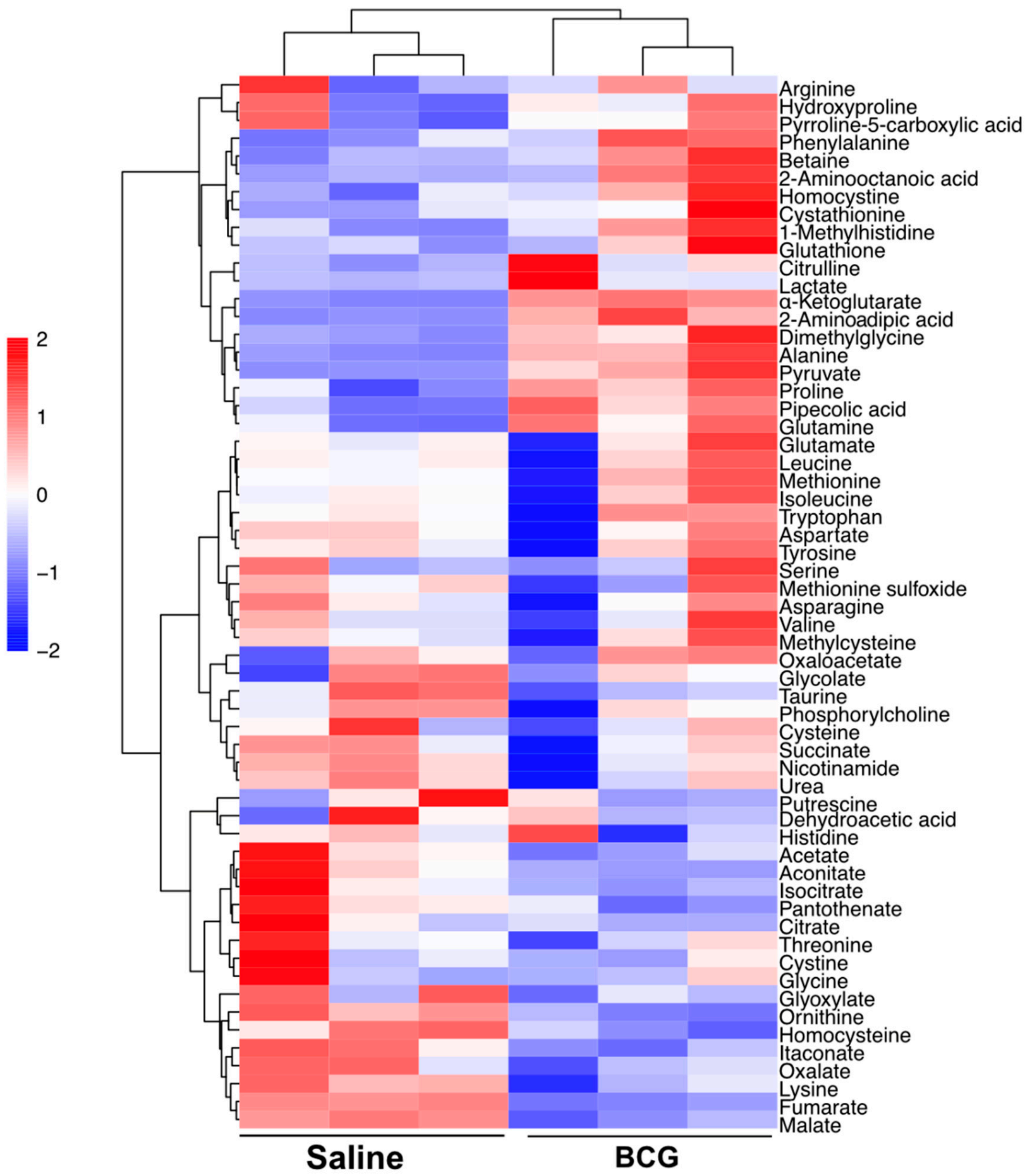

Supplementary Figure S5: Heatmap showing hierarchical clustering of significantly altered metabolites between saline- and BCG-treated groups. Each row represents a metabolite, and each column corresponds to a sample. The color scale indicates relative metabolite abundance (red: higher, blue: lower), normalized across samples. Ward's clustering method was applied to both metabolites and samples, revealing distinct clustering patterns. This analysis highlights marked metabolic differences between the two treatment groups, suggesting that BCG administration induces substantial alterations in metabolic pathways. Group labels are indicated at the bottom of the heatmap, and the clustering dendrogram shows relationships between samples based on metabolic profiles. The dendrogram on the metabolite axis reveals clusters of metabolites that exhibit similar concentration patterns across the samples.

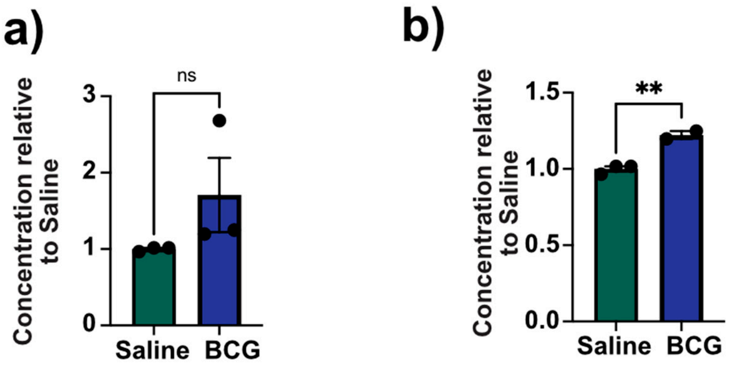

Supplementary Figure S6: Lactate concentration in BMCs from the BCG group. (a) before and (b) after removal of an outlier measurement, identified using Dixon's test (Q = 0.96548, p = 0.05756).

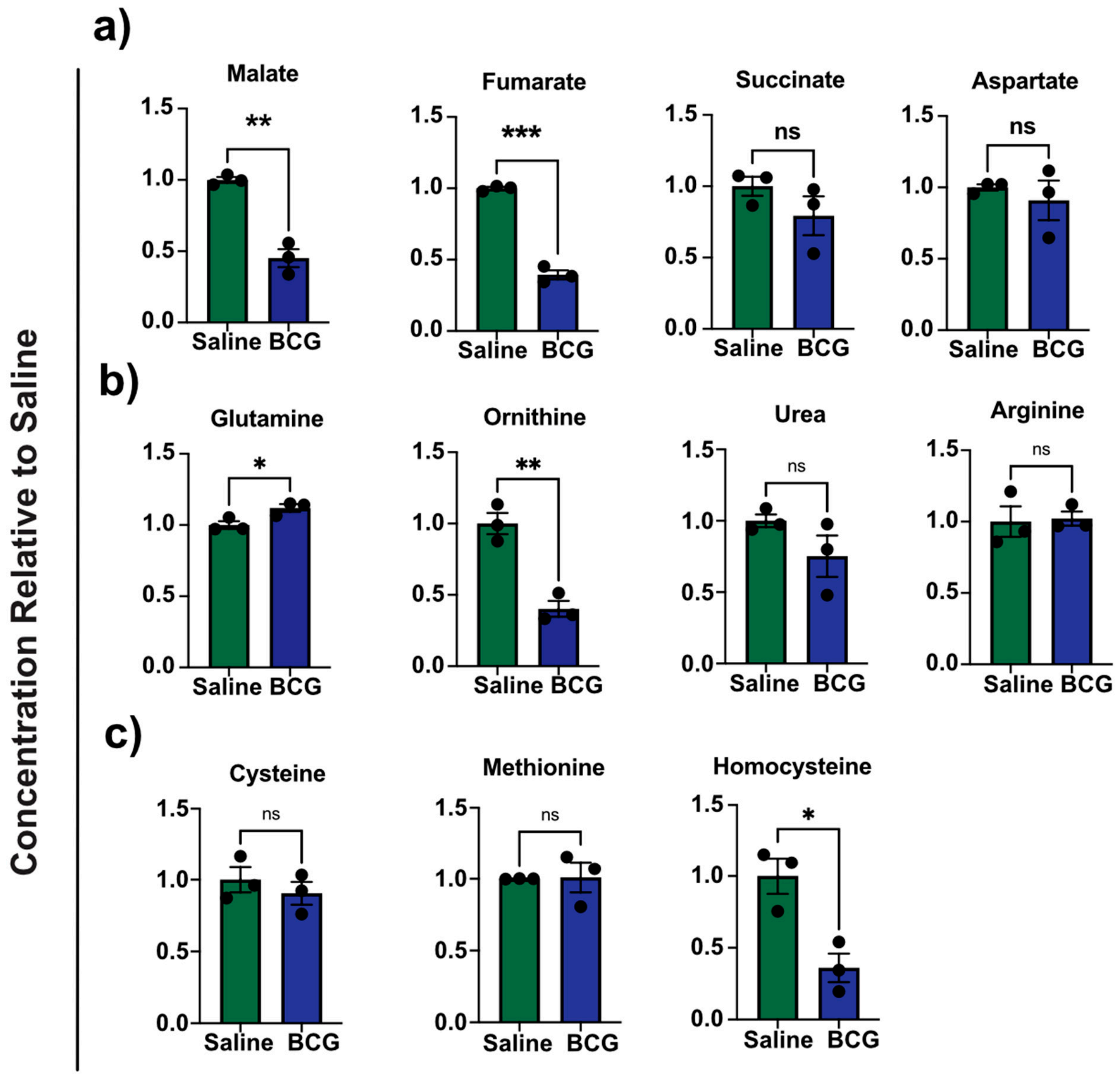

Supplementary Figure S7: Differential levels of metabolites in saline vs. BCG in BMCs. (a) Concentration of metabolites in the TCA cycle, (b) arginine biosynthesis pathway, and (c) cysteine and methionine metabolism pathway. The y-axis indicates relative concentration of metabolites compared with saline, as measured with LC-MS analysis. Data are shown as mean ± SEM; $n = 3$ per group. *$p < 0.05$, **$p < 0.01$, ***$p < 0.001$ by unpaired two-tailed Student's t-test.

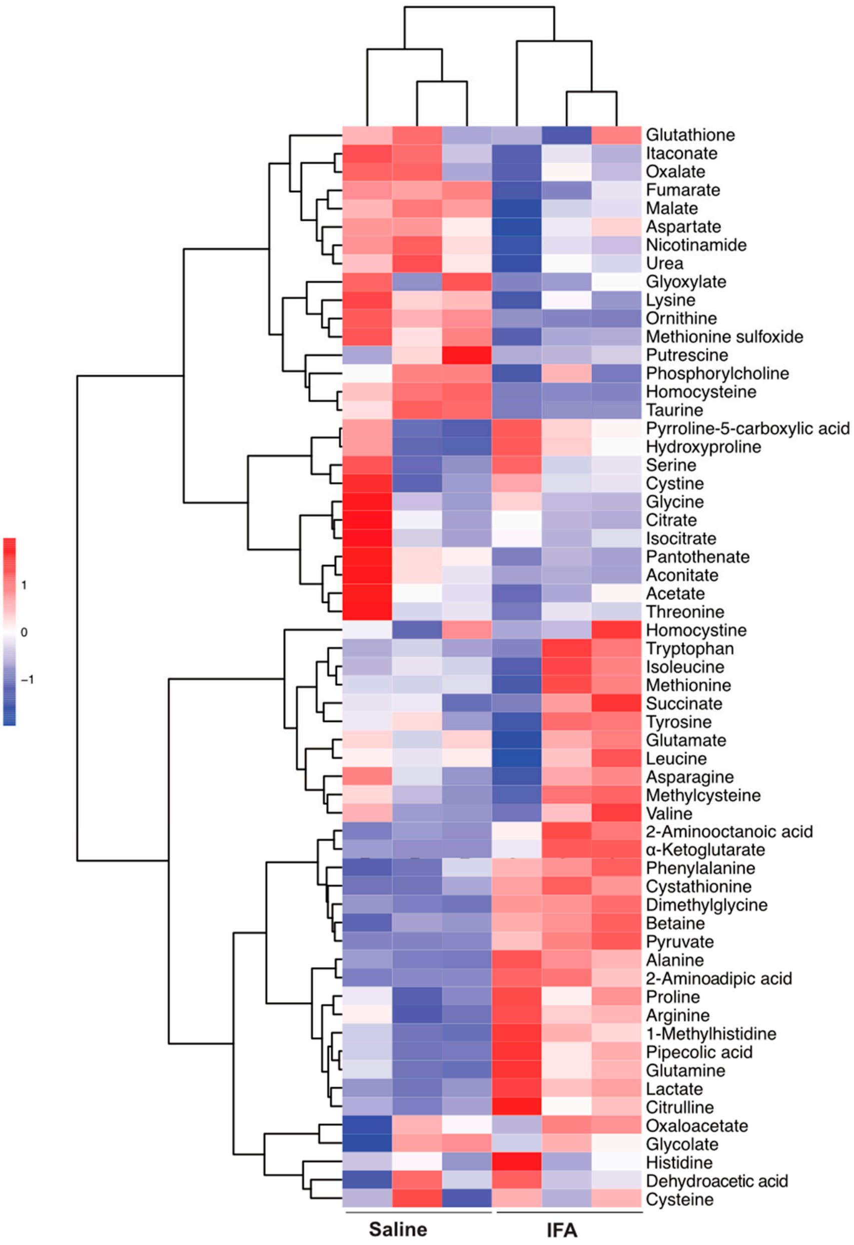

Supplementary Figure S8: Heatmap showing hierarchical clustering of significantly altered metabolites between saline- and IFA-treated groups. Each row represents a metabolite, and each column corresponds to a sample. The color scale indicates relative metabolite abundance (red: higher, blue: lower), normalized across samples. Ward's clustering method was applied to both metabolites and samples, revealing distinct clustering patterns. This analysis highlights marked metabolic differences between the two treatment groups, suggesting that IFA administration induces substantial alterations in metabolic pathways. Group labels are indicated at the bottom of the heatmap, and the clustering dendrogram shows relationships between samples based on metabolic profiles. The dendrogram on the metabolite axis reveals clusters of metabolites that exhibit similar concentration patterns across the samples.

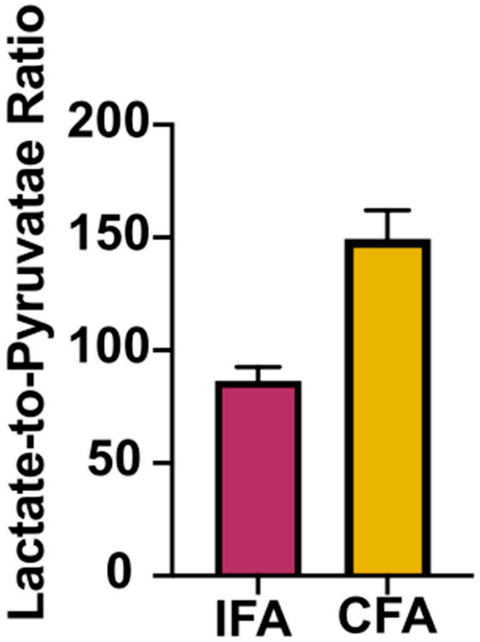

Supplementary Figure S9. Lactate-to-pyruvate ratio in BMCs from mice immunized with IFA or CFA. For each group (n = 3), all possible pairwise combinations of lactate and pyruvate concentrations were used to calculate the lactate-to-pyruvate ratio (3 lactate values × 3 pyruvate values = 9 ratios per group). The mean and standard error of these 9 ratios are shown as mean ± SEM.

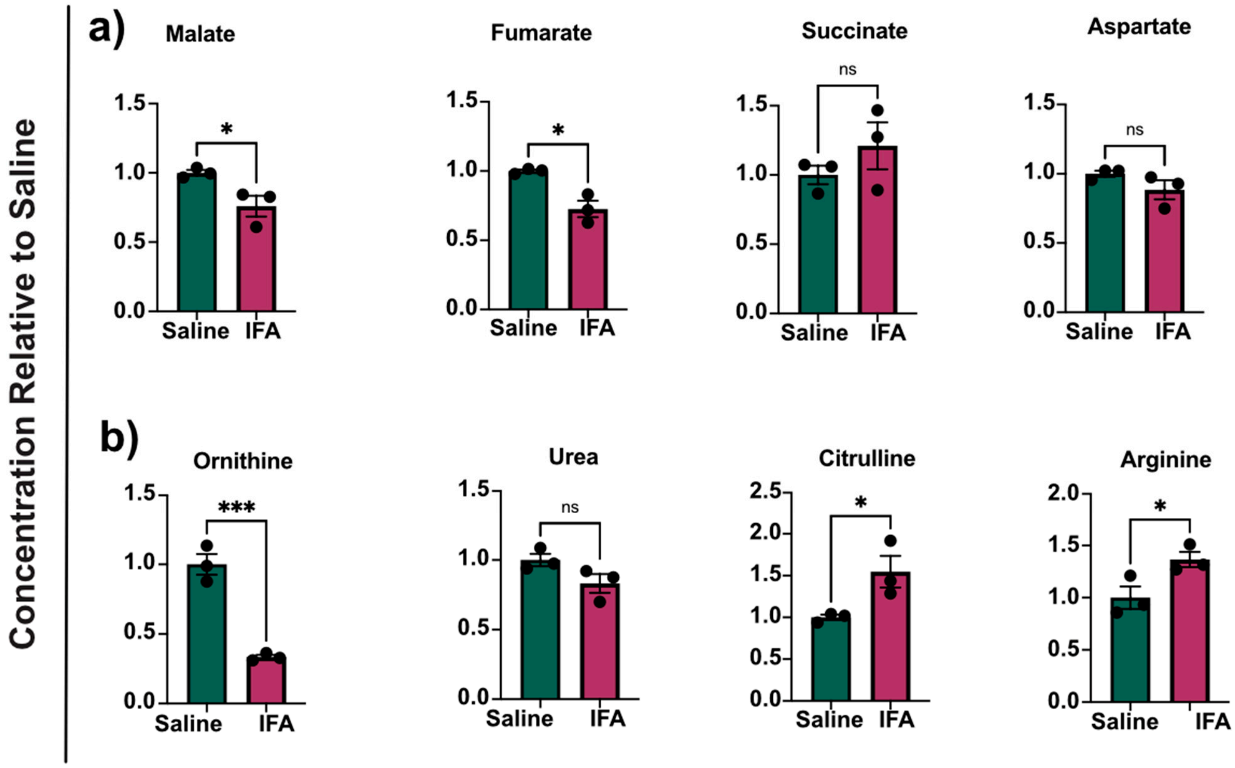

Supplementary Figure S10: Differential level of metabolites in saline vs. IFA in BMCs. (a) Concentration of metabolites in the TCA cycle, and (b) arginine biosynthesis pathway. The y-axis indicates relative concentration of metabolites compared with saline, as measured with LC-MS analysis. Data are shown as mean ± SEM; $n = 3$ per group. $^*p < 0.05$, $^{***}p < 0.001$ by unpaired two-tailed Student's t-test.

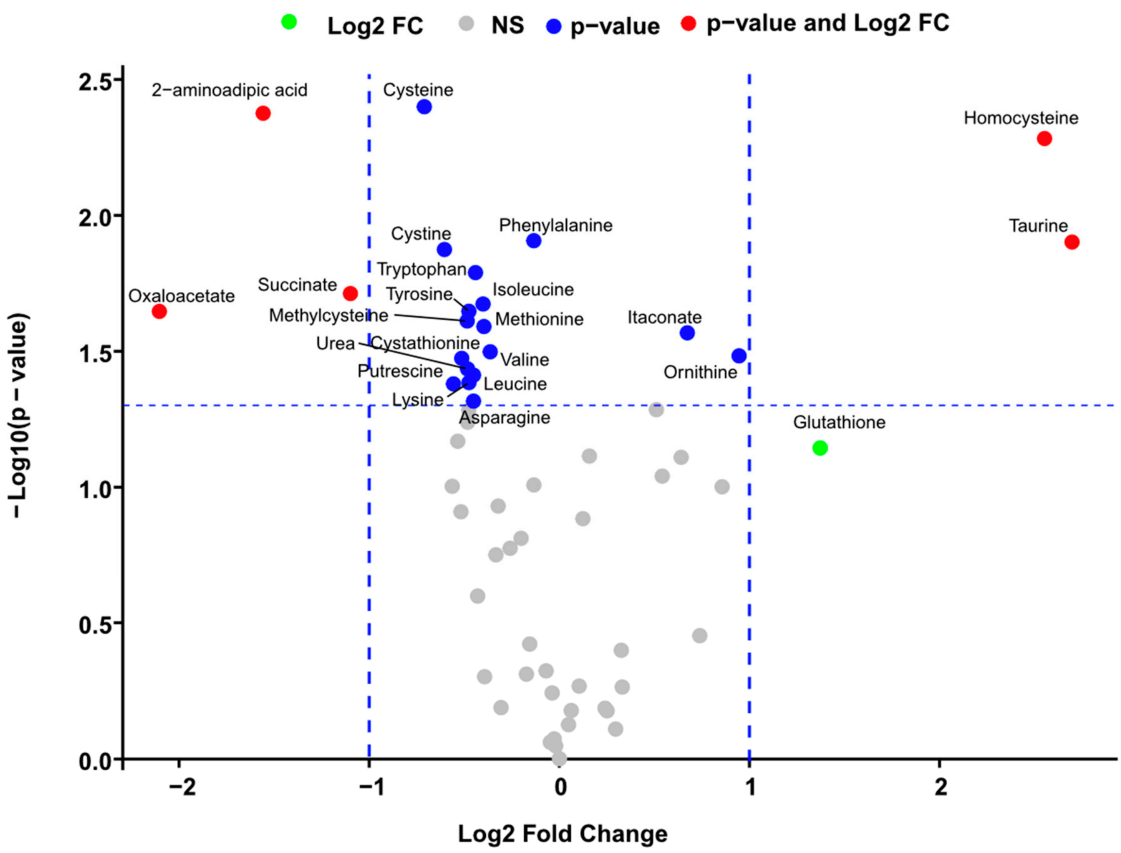

Supplementary Figure S11: Volcano plot illustrating differential metabolite levels between IFA and CFA. The plot shows the relationship between log2 fold change (x-axis) and statistical significance (–log10 *p*-value, y-axis) for individual metabolites. Metabolites with significant fold change and p-value are highlighted in red (*p*-value and Log2 FC), those with significant p-value only are shown in blue, and those with significant Log2 fold change only are shown in green. Non-significant metabolites are shown in grey. Dashed blue lines indicate the thresholds for statistical significance and fold change. Labeled metabolites represent those with the highest significance or fold changes.

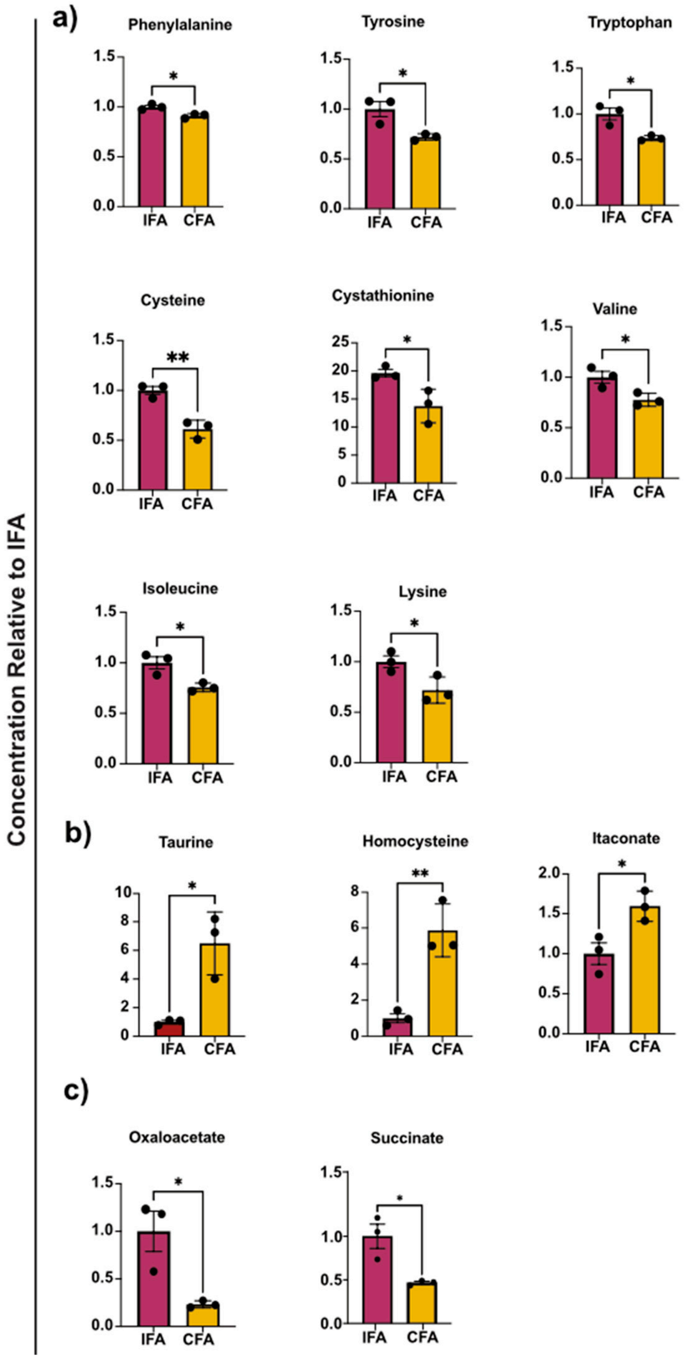

Supplementary Figure S12: Differential level of metabolites in IFA vs. CFA in BMCs. (a) amino acids upregulated in IFA, (b) amino acids and metabolites upregulated in CFA, and (c) TCA cycle intermediates. The y-axis indicates relative concentration of metabolites compared with IFA, as measured with LC-MS analysis. Data are shown as mean ± SEM; $n = 3$ per group. $*p < 0.05$, $**p < 0.01$ by unpaired two-tailed Student's t-test.
