# Supplementary material for: Immunization with Complete Freund’s Adjuvant Reveals Trained Immunity-like Features in A/J Mice
Source: Vaccines (Basel). 2025 Jul 21;13(7):768. doi: 10.3390/vaccines13070768 (PMC12300380; doi:10.3390/vaccines13070768)
Supplement: Supplementary file 1 [file vaccines-13-00768-s001.zip › Mone et al_supplementary tables.pdf]

Supplementary Table S1. Primers used for RT-qPCR.

RT-qPCR primers			
**Target**	**Forward (5' to 3')**	**Reverse (5' to 3')**	**Ref**
**TNF-α**	CTGTGAAGGGAATGGGTGTT	GGTCACTGTCCCAGCATCTT	(1)
**IL-6**	GAGGATACCACTCCCAACAGACC	AAGTGCATCATCGTTGTTCATACA	(2)
**IL-10**	GGTTGCCAAGCCTTATCGGA	ACCTGCTCCACTGCCTTGCT	(2)
**IL-1β**	TGGACCTTCCAGGATGAGGACA	GTTCATCTCGGAGCCTGTAGTG	*
**CCL2**	GCATCCACGTGTTGGCTCA	CTCCAGCCTACTCATTGGGATCA	(3)
**CCL5**	CCTGCTGCTTTGCCTACCTCTC	ACACACTTGGCGGTTCCTTCGA	*
**CXCL10**	GCCGTCATTTTCTGCCTCA	CGTCCTTGCGAGAGGGATC	(4)
**F4/80**	CGTGTTGTTGGTGGCACTGTGA	CCACATCAGTGTTCCAGGAGAC	*
**PPARγ**	CGGTTTCAGAAGTGCCTTG	GGTTCAGCTGGTCGATATCAC	(5)
**CD206**	GTTCACCTGGAGTGATGGTTCTC	AGGACATGCCAGGGTCACCTTT	*
**NOS2**	GGCAGCCTGTGAGACCTTT	TTGGAAGTGAAGCGTTTCG	(1)
**CD80**	CCTCAAGTTTCCATGTCCAAGGC	GAGGAGAGTTGTAACGGCAAGG	*
**CD86**	ACGTATTGGAAGGAGATTACAGCT	TCTGTCAGCGTTACTATCCCGC	*
**GAPDH**	CTCCCACTCTTCCACCTTCG	GCCTCTCTTGCTCAGTGTCC	(6)

*Primer sequences were obtained from publicly accessible commercial resources and verified target specificity independently using NCBI Primer-BLAST.

Supplementary Table S2. Primers used for ChIP-qPCR.

ChIP-qPCR primers			
**Target**	**Forward (5' to 3')**	**Reverse (5' to 3')**	**Ref**
**TNF-α**	CACCCCGAAGTTCAGTAGACA	GAACTGGCAGAAGAGGCACT	(7)
**IL-6**	TTTCTCCACGCAGGAGACTT	TCCACGATTTCCCAGAGAAC	(7)
**IL-10**	GTGGCTATCACCGTGCAGTA	AAGCAACTGCCTCTCTGAGC	(7)
**IL-1β**	TTAGCATGCCTGCCCTGAAC	GGTGGGCATCCAGCGTTA	(8)
**GAPDH**	ATCCTGTAGGCCAGGTGATG	AGGCTCAAGGGCTTTTAAGG	(9)

Supplementary Table S3. List of metabolites detected and quantified using targeted LC-MS analysis. The table indicates metabolites, HMDB ID (10), and the major pathways involved.

No	Metabolites	HMDB ID	Major pathways involved
1.	1-Methylhistidine	HMDB0000001	Histidine metabolism
2.	2-Aminoadipic acid	HMDB0302754	Lysine biosynthesis
3.	2-Aminooctanoic acid	HMDB0000991	-
4.	Acetate	HMDB0000042	Glycolysis and gluconeogenesis
5.	Aconitate	HMDB0000072	TCA cycle
6.	Alanine	HMDB0000161	Alanine, aspartate and glutamate metabolism
7.	α-ketoglutarate	HMDB0000208	TCA cycle
8.	Arginine	HMDB0000517	Arginine biosynthesis
9.	Asparagine	HMDB0000168	Alanine, aspartate and glutamate metabolism
10.	Aspartate	HMDB0000191	Arginine biosynthesis
11.	Betaine	HMDB0000043	Glycine, serine and threonine metabolism
12.	Citrate	HMDB0000094	TCA cycle
13.	Citrulline	HMDB0000904	Arginine biosynthesis
14.	Cystathionine	HMDB0000099	Transsulfuration pathway
15.	Cysteine	HMDB0000574	Glycine, serine and threonine metabolism
16.	Cystine	HMDB0250712	Cysteine and methionine metabolism
17.	Dehydroacetic acid	HMDB0250943	-
18.	Dimethylglycine	HMDB0000092	Glycine, serine and threonine metabolism
19.	Fumarate	HMDB0000134	TCA cycle
20.	Glutamate	HMDB0000148	Arginine biosynthesis
21.	Glutamine	HMDB0000641	Arginine biosynthesis
22.	Glutathione	HMDB0000125	Cysteine and Methionine metabolism
23.	Glycine	HMDB0000123	Purine metabolism
24.	Glycolate	HMDB0000115	Glyoxylate and dicarboxylate metabolism
25.	Glyoxylate	HMDB0000119	Glyoxylate and dicarboxylate metabolism
26.	Histidine	HMDB0000177	Histidine metabolism
27.	Homocysteine	HMDB0000742	Cysteine and methionine metabolism
28.	Homocystine	HMDB0000575	Cysteine and methionine metabolism
29.	Hydroxyproline	HMDB0000725	Arginine and proline metabolism
30.	Isocitrate	HMDB0000193	TCA cycle
31.	Isoleucine	HMDB0000172	Valine, leucine and isoleucine metabolism
32.	Itaconate	HMDB0002092	TCA cycle
33.	Lactate	HMDB0000190	Glycolysis and gluconeogenesis

34.	Leucine	HMDB0000687	Valine, leucine and isoleucine metabolism
35.	Lysine	HMDB0000182	Lysine biosynthesis and degradation
36.	Malate	HMDB0000156	TCA cycle
37.	Methionine	HMDB0000696	Cysteine and methionine metabolism
38.	Methionine sulfoxide	HMDB0002005	-
39.	Methylcysteine	HMDB0002108	-
40.	Nicotinamide	HMDB0001406	Nicotinate and nicotinamide metabolism
41.	Ornithine	HMDB0000214	Arginine and proline metabolism
42.	Oxalate	HMDB0002329	Glyoxylate and dicarboxylate metabolism
43.	Oxaloacetate	HMDB0000223	Glycolysis and gluconeogenesis
44.	Pyrroline-5-carboxylate	HMDB0001301	Alanine, aspartate and glutamate metabolism
45.	Pantothenate	HMDB0000210	beta-Alanine metabolism
46.	Phenylalanine	HMDB0000159	Phenylalanine metabolism
47.	Phosphorylcholine	HMDB0001565	Glycerophospholipid metabolism
48.	Pipecolic acid	HMDB0000070	Lysine degradation
49.	Proline	HMDB0000162	Arginine and proline metabolism
50.	Putrescine	HMDB0001414	Arginine and proline metabolism
51.	Pyruvate	HMDB0000243	Glycolysis and gluconeogenesis
52.	Serine	HMDB0000187	Glycine, serine and threonine metabolism
53.	Succinate	HMDB0000254	TCA cycle
54.	Taurine	HMDB0000251	Taurine and hypotaurine metabolism
55.	Threonine	HMDB0000167	Glycine, serine and threonine metabolism
56.	Tryptophan	HMDB0000929	Tryptophan metabolism
57.	Tyrosine	HMDB0000158	Tyrosine metabolism
58.	Urea	HMDB0000294	Arginine biosynthesis
59.	Valine	HMDB0000883	Valine, leucine and isoleucine metabolism

Supplementary Table S4: List of metabolites significantly altered in the CFA group compared to the saline group, used to identify major pathways affected by CFA immunization. Statistical significance was determined using Student's *t*-test with a *p*-value cutoff of 0.05.

No	Metabolite	HMDB ID	Pattern of changes in the CFA group relative to the Saline group
1.	1-Methylhistidine	HMDB0000001	↑
2.	2-Aminoadipic acid	HMDB0302754	↑
3.	2-Aminooctanoic acid	HMDB0000991	↑
4.	α-ketoglutarate	HMDB0000208	↑
5.	Acetate	HMDB0000042	↓
6.	Alanine	HMDB0000161	↑
7.	Asparagine	HMDB0000168	↓
8.	Aspartate	HMDB0000191	↓
9.	Cysteine	HMDB0000574	↓
10.	Fumarate	HMDB0000134	↓
11.	Homocysteine	HMDB0000742	↓
12.	Isoleucine	HMDB0000172	↓
13.	Lactate	HMDB0000190	↑
14.	Leucine	HMDB0000687	↓
15.	Lysine	HMDB0000182	↓
16.	Malate	HMDB0000156	↓
17.	Methionine	HMDB0000696	↓
18.	Methionine sulfoxide	HMDB0002005	↓
19.	Methylcysteine	HMDB0002108	↓
20.	Nicotinamide	HMDB0001406	↓
21.	Ornithine	HMDB0000214	↓
22.	Proline	HMDB0000162	↑
23.	Pyruvate	HMDB0000243	↑
24.	Succinate	HMDB0000254	↓
25.	Tryptophan	HMDB0000929	↓
26.	Tyrosine	HMDB0000158	↓
27.	Urea	HMDB0000294	↓
28.	Valine	HMDB0000883	↓

Supplementary Table S5: List of metabolites significantly altered in the BCG group compared to the saline group, used to identify major pathways affected by BCG immunization. Statistical significance was determined using Student's *t*-test with a *p*-value cutoff of 0.05.

No	Metabolite	HMDB ID	Pattern of changes in the BCG group relative to the Saline group
1.	2-Aminoadipic acid	HMDB0302754	↑
2.	Alanine	HMDB0000161	↑
3.	α-Ketoglutarate	HMDB0000208	↑
4.	Dimethylglycine	HMDB0000092	↑
5.	Fumarate	HMDB0000134	↓
6.	Glutamine	HMDB0000641	↑
7.	Homocysteine	HMDB0000742	↓
8.	Itaconate	HMDB0002092	↓
9.	Lysine	HMDB0000182	↓
10.	Malate	HMDB0000156	↓
11.	Ornithine	HMDB0000214	↓
12.	Pipecolic acid	HMDB0000070	↑
13.	Proline	HMDB0000162	↑
14.	Pyruvate	HMDB0000243	↑
15.	Lactate	HMDB0000190	↑
16.	Taurine	HMDB0000251	↓

Supplementary Table S6: List of metabolites significantly altered in the IFA group compared to the saline group, used to identify major pathways affected by IFA immunization. Statistical significance was determined using Student's *t*-test with a *p*-value cutoff of 0.05.

No	Metabolite	HMDB ID	Pattern of changes in the IFA group relative to the Saline group
1.	1-Methylhistidine	HMDB0000001	↑
2.	2-Aminoadipic acid	HMDB0302754	↑
3.	2-Aminooctanoic acid	HMDB0000991	↑
4.	α-ketoglutarate	HMDB0000208	↑
5.	Alanine	HMDB0000161	↑
6.	Arginine	HMDB0000517	↑
7.	Betaine	HMDB0000043	↑
8.	Citrulline	HMDB0000904	↑
9.	Cystathionine	HMDB0000099	↑
10.	Dimethylglycine	HMDB0000092	↑
11.	Fumarate	HMDB0000134	↓
12.	Glutamine	HMDB0000641	↑
13.	Homocysteine	HMDB0000742	↓
14.	Lactate	HMDB0000190	↑
15.	Lysine	HMDB0000182	↓
16.	Malate	HMDB0000156	↓
17.	Methionine sulfoxide	HMDB0002005	↓
18.	Nicotinamide	HMDB0001406	↓
19.	Ornithine	HMDB0000214	↓
20.	Phenylalanine	HMDB0000159	↑
21.	Pipecolic acid	HMDB0000070	↑
22.	Proline	HMDB0000162	↑
23.	Pyruvate	HMDB0000243	↑
24.	Taurine	HMDB0000251	↓

Supplementary Table S7: Major findings of the TI features associated with CFA immunization.

Parameter	Findings
Cytokines	Upregulation of the expression of inflammatory cytokines (TNF-α, IL-6, IL-1β, and IFN-β), and chemokines (CCL5, and CXCL10)
Metabolomics	Rewiring of glycolysis (↑ lactate, pyruvate, and alanine), TCA cycle (↓ fumarate, malate and succinate), and amino acid metabolism (↓ methionine, cysteine, and homocysteine)
Epigenetics	Histone enrichment in the promoter region of TNF-α

REFERENCES

1. Jablonski KA, Gaudet AD, Amici SA, Popovich PG, Guerau-de-Arellano M. 2016. **Control of the Inflammatory Macrophage Transcriptional Signature by miR-155**. PLoS One 11:e0159724.
2. Overbergh L, Giulietti A, Valckx D, Decallonne R, Bouillon R, Mathieu C. 2003. **The use of real-time reverse transcriptase PCR for the quantification of cytokine gene expression**. J Biomol Tech 14:33-43.
3. Ishida Y, Kimura A, Kuninaka Y, Inui M, Matsushima K, Mukaida N, Kondo T. 2012. **Pivotal role of the CCL5/CCR5 interaction for recruitment of endothelial progenitor cells in mouse wound healing**. J Clin Invest 122:711-21.
4. Ozga AJ, Chow MT, Lopes ME, Servis RL, Di Pilato M, Dehio P, Lian J, Mempel TR, Luster AD. 2022. **CXCL10 chemokine regulates heterogeneity of the CD8(+) T cell response and viral set point during chronic infection**. Immunity 55:82-97 e8.
5. Fujiki K, Kano F, Shiota K, Murata M. 2009. **Expression of the peroxisome proliferator activated receptor gamma gene is repressed by DNA methylation in visceral adipose tissue of mouse models of diabetes**. BMC Biol 7:38.
6. Ruiz-Villalba A, Mattiotti A, Gunst QD, Cano-Ballesteros S, van den Hoff MJ, Ruijter JM. 2017. **Reference genes for gene expression studies in the mouse heart**. Sci Rep 7:24.
7. Jeljeli M, Riccio LGC, Doridot L, Chene C, Nicco C, Chouzenoux S, Deletang Q, Allanore Y, Kavian N, Batteux F. 2019. **Trained immunity modulates inflammation-induced fibrosis**. Nat Commun 10:5670.
8. Schaafsma W, Zhang X, van Zomeren KC, Jacobs S, Georgieva PB, Wolf SA, Kettenmann H, Janova H, Saiepour N, Hanisch UK, Meerlo P, van den Elsen PJ, Brouwer N, Boddeke HW, Eggen BJ. 2015. **Long-lasting pro-inflammatory suppression of microglia by LPS-preconditioning is mediated by RelB-dependent epigenetic silencing**. Brain Behav Immun 48:205-21.
9. Su H, Liang Z, Weng S, Sun C, Huang J, Zhang T, Wang X, Wu S, Zhang Z, Zhang Y, Gong Q, Xu Y. 2021. **miR-9-5p regulates immunometabolic and epigenetic pathways in beta-glucan-trained immunity via IDH3alpha**. JCI Insight 6.
10. Wishart DS, Feunang YD, Marcu A, Guo AC, Liang K, Vazquez-Fresno R, Sajed T, Johnson D, Li C, Karu N, Sayeeda Z, Lo E, Assempour N, Berjanskii M, Singhal S, et al. 2018. **HMDB 4.0: the human metabolome database for 2018**. Nucleic Acids Res 46:D608-D617.
